# Patterns of homoeologous gene expression shown by RNA sequencing in hexaploid bread wheat

**DOI:** 10.1186/1471-2164-15-276

**Published:** 2014-04-11

**Authors:** Lindsey J Leach, Eric J Belfield, Caifu Jiang, Carly Brown, Aziz Mithani, Nicholas P Harberd

**Affiliations:** 1 Department of Plant Sciences, University of Oxford, Oxford, UK; 2 School of Biosciences, University of Birmingham, Birmingham, UK; 3 Syed Babar Ali School of Science and Engineering, Lahore University of Management Sciences, Lahore, Pakistan

**Keywords:** Wheat, Wheat transcriptome, mRNA-Seq, Diploidization, Homoeologues, Polyploidy

## Abstract

**Background:**

Bread wheat (*Triticum aestivum*) has a large, complex and hexaploid genome consisting of A, B and D homoeologous chromosome sets. Therefore each wheat gene potentially exists as a trio of A, B and D homoeoloci, each of which may contribute differentially to wheat phenotypes. We describe a novel approach combining wheat cytogenetic resources (chromosome substitution ‘nullisomic-tetrasomic’ lines) with next generation deep sequencing of gene transcripts (RNA-Seq), to directly and accurately identify homoeologue-specific single nucleotide variants and quantify the relative contribution of individual homoeoloci to gene expression.

**Results:**

We discover, based on a sample comprising ~5-10% of the total wheat gene content, that at least 45% of wheat genes are expressed from all three distinct homoeoloci. Most of these genes show strikingly biased expression patterns in which expression is dominated by a single homoeolocus. The remaining ~55% of wheat genes are expressed from either one or two homoeoloci only, through a combination of extensive transcriptional silencing and homoeolocus loss.

**Conclusions:**

We conclude that wheat is tending towards functional diploidy, through a variety of mechanisms causing single homoeoloci to become the predominant source of gene transcripts. This discovery has profound consequences for wheat breeding and our understanding of wheat evolution.

## Background

Whole genome duplication (WGD) by polyploidy is a major driving force in the evolution of eukaryotes, particularly plants and fungi [1,2]. All flowering plants have undergone at least one round of WGD [3]. Between 30-80% of species are currently polyploids [4], while the rest exist as paleopolyploids [5,6], having undergone a gradual process of “diploidization,” or reversion to a diploid state over evolutionary time. This process involves extensive genomic rearrangements, including the physical loss of a large fraction of duplicate regions, and the accumulation of mutations that distinguish the sequences of the duplicate ‘homoeologous’ copies and contribute to their functional divergence [7-9].

Many of our most important crop species are currently either autopolyploid, for example potato and coffee, or allopolyploid, for example pasta and bread wheats, oat, cotton and canola. Allopolyploid plants often undergo major changes in genome structure and function induced by the “genomic shock” [10,11] occurring when two divergent genomes combine. For example, newly formed synthetic wheats undergo changes in DNA methylation and rapid, non-random elimination of up to 14% of genomic DNA sequence, which differentiates the homoeologous chromosomes and fosters the transition to diploid-like meiotic behaviour [12-15]. In addition, newly formed hexaploid wheat lines are frequently unstable, leading to aneuploid plants with missing or extra chromosomes [16]. Other major crops are paleopolyploids, including barley, maize and rice, whose diploid genomes have been reduced towards their ancestral chromosome number and gene content through removal of duplicate copies, called homoeologues or homoeoloci, in a process of fractionation. Far from being a random process, genes are commonly removed preferentially from particular homoeologues, in a process termed “biased fractionation” [17]. This bias in gene densities between homoeologous subgenomes has been observed in several paleopolyploid plants including *Arabidopsis*[18], maize [19], Brassica [20] and soybean [21,22].

A hallmark feature of polyploid genomes is rapid and novel change in gene expression, ranging from slight alterations in the expression of homoeoloci through to the complete absence of expression caused by epigenetic homoeolocus silencing [23,24]. Studies of both natural and synthetic polyploids have shown that homoeoloci frequently make unequal contributions to total gene expression levels. For example, they may become differentially regulated in a tissue-specific manner [25,26], in response to stress [27], during development [28] or during different stages of fermentation in yeast [29]. In allotetraploid plants of various ages, genes from one homoeologous subgenome, typically the less fractionated subgenome, make a greater contribution to the transcriptome, a phenomenon termed “genome dominance”. These include newly synthesized *Arabidopsis suecica*[30], the natural allotetraploid *Tragopogon miscellus* that originated within the last 80 years [31], and allotetraploid cotton [32,33], originating naturally 1–2 million years ago or synthetic (newly synthesized) [34]. Genome dominance has also been observed in autotetraploid maize [35], even though polyploidization occurred some 12 million years ago. However, it has not been observed in the more ancient plant tetraploidies, such as those in the *Arabidopsis* or rice lineages [6,36].

Bread wheat (*Triticum aestivum*) is an excellent model system for studying the long-term responses of plant genomes to polyploidy. It is also a vital crop to food security, accounting for almost 20% of the world’s daily food consumption [37]. Hexaploid bread wheat evolved through two distinct interspecies hybridization steps, involving three diploid donor species each with 7 pairs of chromosomes. First, ~0.5 million years ago, tetraploid *T. turgidum* arose from hybridization between a wild diploid species closely related to extant *T. urartu* (AA chromosomes) and another unknown wild diploid species related to *Aegilops speltoides*[38], called the B genome donor. The *T. turgidum* genome is symbolized by AABB. About 8,500 years ago, hexaploid *T. aestivum* (AABBDD) arose from hybridization between cultivated *T. turgidum* and the wild diploid species *A. tauschii* (DD). The 42 chromosomes of the large ~17 Gb bread wheat genome [39,40] are therefore distributed in three diploid homoeologous sets (A, B and D), each comprising seven chromosome pairs. Within each chromosomal group (e.g., group 1), each diploid chromosome pair (e.g., the 1A pair) is largely homozygous due to the natural inbreeding of the polyploid wheats and does not recombine with its homoeologues (e.g., the 1B and 1D pairs) [41]. Thus, every bread wheat gene is potentially represented by at least six copies; there are three homoeoloci (or homoeologues) carried on the three (A, B and D) homoeologous chromosome pairs, with each homoeolocus representing a diploid pair of homoeoloci.

Since the diploid progenitors of wheat originate from a common ancestor, each trio of A, B and D homoeoloci in hexaploid wheat originates from a common ancestral gene. Hence a simple null hypothesis would be equal expression of each homoeolocus, with A, B and D homoeoloci contributing mRNA transcripts (‘A = B = D’). There are two main reasons why deviations from this 1:1:1 ratio might be widespread. First, evolutionary divergence of homoeoloci may have caused changes in relative expression, from slight changes, to physical loss (deletion) of a homoeolocus. Second, the complex successive hybridization and domestication processes that have shaped bread wheat may have influenced homoeolocus divergence, in particular the coordinate regulation of relative homoeolocus expression levels. In synthetic hexaploid wheat, differential expression of homoeoloci is rapidly established [42,43], is stably inherited [44] and is similar to the patterns observed in natural hexaploids [44]. Some studies have also showed a parent-specific expression dominance in the synthetic allohexaploid wheat transcriptome, involving a biased contribution from the AB (tetraploid) genome parent [43,45], though others have not detected any genome dominance [44]. These studies have been somewhat limited by the inability to discriminate gene expression from all three individual (A, B and D) homoeoloci. While the differential contribution from all three homoeoloci has been illustrated for a small number of individual genes [46-48], little is known about the relative contributions of individual homoeoloci to wheat phenotypes on a larger scale, or about the existence of any discernible genome-wide patterns.

Here we systematically determine the extent to which bread wheat genes retained in three homoeologous copies deviate from the null hypothesis of equal expression. This determination presents an intricate challenge. Distinguishing expression of a specific homoeolocus from expression of other similar sequences is difficult, in part because the sequences of homoeoloci are very similar [40]. The wheat genome is also highly repetitive, having more than 80% repetitive DNA [49,50]. So, in addition to three potential homoeologous copies of each gene, there may be multiple “paralogues,” here defined as similar loci resulting from gene duplication. Such paralogues may be present on more than one chromosomal group (e.g., on both the group 1 and group 5 chromosome groups). Several strategies have been employed to assemble homoeologue-specific bread wheat sequences. These include approaches that reduce the sequence complexity, including the physical isolation and sequencing of specific chromosome homoeologues [51-54], direct assembly of transcriptomic sequences [55] and utilizing the sequence similarity between bread wheat subgenomes and those of its diploid progenitors [40,56]. In a recent landmark study, Brenchley et al. [40] accomplished direct gene assembly from wheat genomic sequences and assigned two thirds of gene sequences to their homoeologous chromosomes, based on sequence similarity with diploid species related to the progenitors of the A, B and D subgenomes.

Here we describe a novel approach to understanding wheat gene expression patterns that combines wheat cytogenetic resources with next generation RNA sequencing. This approach enables assessment of homoeologous gene expression for a sample of ~2,300 genes located on the group 1 and 5 chromosome groups. Our study shows detailed global patterns of relative homoeologous contribution through homoeolocus-specific characterization of bread wheat gene transcripts. In particular, we find that reduction in complexity, through the mechanisms of homoeolocus loss, silencing and biased expression, is a major feature of wheat genome evolution.

## Results

### Alignment of reads from euploid and nullitetra wheat cDNA libraries to a partial reference transcriptome

**Figure 1:**
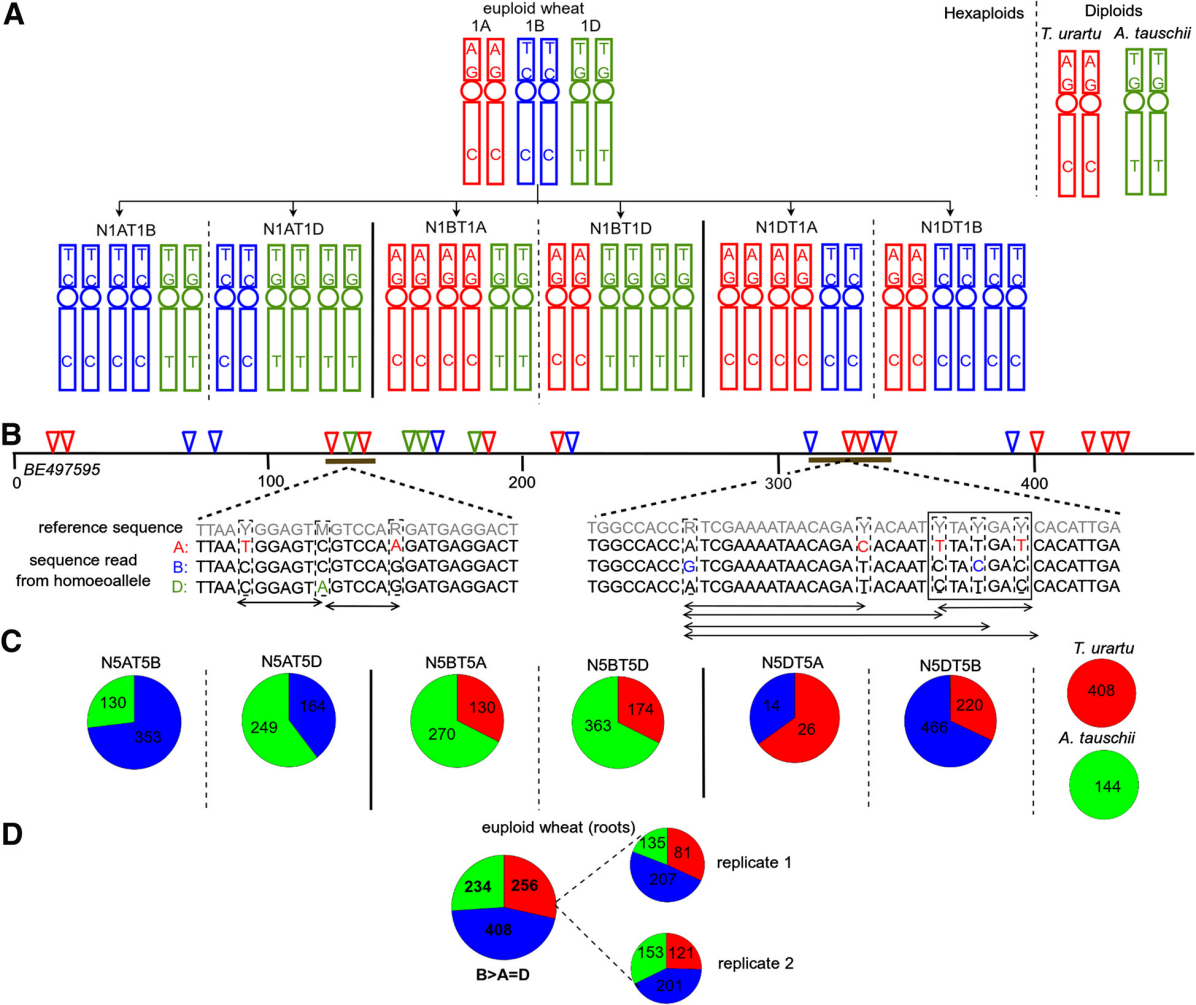
**Analysis of hexaploid bread wheat homoeologous gene expression using nullisomic-tetrasomic lines. (A)** Schematic illustrating the group 1 homoeologous chromosomes in hexaploid bread wheat, the derived nullisomic-tetrasomic lines and the A and D genome diploids. A single homoeologue-specific variant (HSV) diagnostic for each of A (red), B (blue) and D (green) homoeologous chromosomes is shown. **(B)** Two sets of three homoeologue-specific haplotypes of *BE497595*, a gene encoding a chloroplast thylakoidal peptidase-like protein and which has 24 HSVs (inverted triangles) diagnostic for either 5A (red), 5B (blue) or 5D (green) homoeologues. RNA-Seq reads are aligned to a degenerate reference sequence shown in grey containing genetic ambiguity codes at HSV locations. Double ended arrows illustrate candidate sequence regions for haplotype quantification. **(C,D)** RNA-Seq read frequencies from roots for the nullitetras and A and D genome diploids **(C)** and in two biological replicates of euploid wheat **(D)** for each homoeologue-specific haplotype given in the boxed region shown in **(B)**. The homoeologous expression pattern in euploid wheat roots is indicated below the pie chart.

The hexaploid nature of the wheat genome permits the engineering of wheat lines lacking specific homoeologous chromosomes, lines known as nullisomic-tetrasomics (‘nullitetras’) [57,58]. In each nullitetra line, lack (‘nulli’) of one homoeologue is compensated by an extra set of either of the remaining homoeologous chromosomes, thus restoring the hexaploid state. For example, N1AT1B lacks chromosome 1A but has two sets (‘tetra’) of chromosome 1B (Figure 1A). The nullitetras have historically been used for determining presence of wheat genes on specific homoeologous chromosomes [59-61], and we reasoned that, in combination with next generation sequencing, they could be used to develop a systematic understanding of the relative contributions of individual homoeoloci to overall wheat gene expression.

We therefore obtained mRNA-Seq datasets [62] from non-normalized cDNA libraries created from shoot and root tissues of the euploid bread wheat cultivar Chinese Spring, from which the nullitetra lines are derived [58], from complete sets of chromosome 1 and 5 nullitetras, and from extant relatives of the diploid A (*Triticum urartu*) and D (*Aegilops tauschii*) genome donors, herein referred to as A and D genome diploids (Figure 1A; see Additional file 1: Table S1). In the then absence of a *T. aestivum* genome sequence, we exploited an extensive wheat ‘expressed sequence tags’ (ESTs) resource to construct a reference transcriptome sequence (see Additional file 2: Table S2). We selected ESTs physically mapped to segments (‘bins’) of wheat group 1 or group 5 chromosomes [58,63]. Concatenating ESTs from chromosomes 1 (1,123) and 5 (1,247), we created a wheat group 1 and 5 partial reference transcriptome consisting of 2,354 ESTs (see Methods). Since the mean length of these ESTs (hereafter referred to as ‘genes’) was 455 ± 133 bp (see Additional file 3: Figure S1), the genes in our reference are representative of fragments of sequence transcribed from entire genes in their subgenome of origin. Assuming the diploid wheat genome contains approximately ~32,000 genes [40], this reference set represents ~5-10% of the total wheat gene content.

We next aligned our wheat RNA-Seq reads using novoalign software [64] to the group 1 and 5 reference transcriptome, using a number of quality filters (see Additional file 4: Figure S2) to give high confidence read alignments, with a deep average coverage of 700x enabling gene expression analysis (Table 1). On average these reads represented 5.2 ± 1.3% of the total reads from each sample (see Additional file 1: Table S1), a figure roughly comparable with the above estimation that the group 1 and 5 reference transcriptome represents ~5-10% of wheat genes.

**Table 1:** Summary statistics for homoeologue-specific variants (HSVs) in euploid wheat

**Chrom group**	**Reference transcriptome length (bp)**	**RNA-Seq coverage**^**a**^	**Number (%) of 2-allele HSVs diagnostic for homoeolocus**			**P value**^**b**^	**Number (%) of 3-allele HSVs**	**Total HSVs**	**HSV density**^**c**^
			A	B	D				
1	505,132	540 X	2,611	2,830	1,883	1.89x10^-44^	97	7,421	0.015
			(35.2)	(38.1)	(25.4)		(1.3)		
5	567,044	860 X	2,905	2,837	2,710	0.031	94	8,546	0.015
			(34.0)	(33.2)	(31.7)		(1.1)		
Total	1,072,176	700 X	5,516	5,667	4,593	1.21x10^-28^	191	15,776	0.015
			(34.5)	(35.5)	(28.8)		(1.2)		

^a^Coverage is given as the average number of quality filtered reads aligned per site using novoalign software, from all euploid Chinese Spring wheat samples.

^b^*p* for a chi-square test of equal numbers of 2-allele HSVs diagnostic for each homoeolocus.

^c^HSV density is given as the average number of variants per site.

### Homoeolocus-specific sequence analysis shows at least 45% of wheat genes are expressed from all three homoeoloci

We next developed a novel strategy for the direct detection of wheat sequence variants and their assignment to homoeologous chromosomes using nullitetra analysis (see Additional file 4: Figure S2). We implemented this strategy computationally using Perl and visually using the Integrative Genomics Viewer [65]. Homoeologue-specific variants (HSVs) are defined as single base differences between homoeolocus sequences, and candidate HSVs were therefore identified at nucleotide positions (‘sites’) in the reference sequence where 2 or 3 different bases were detected in aligned euploid wheat reads. True HSVs that were ‘diagnostic’ (i.e., specific) for a particular homoeologue were confirmed by the presence of the diagnostic base at a particular site in all nullitetra lines except for those lines lacking the corresponding homoeologous chromosome. For example, Figure 1A illustrates a notional ‘A/T’ homoeologue-specific variant at the tip of the short arm of chromosome 1, where the A base is diagnostic for the A homoeologue, as distinct from the T base present on the B and D homoeologues. Such ‘diagnostic’ sequences were used to infer the expression (and therefore presence) of a particular gene on a specific homoeologous chromosome.

**Figure 2:**
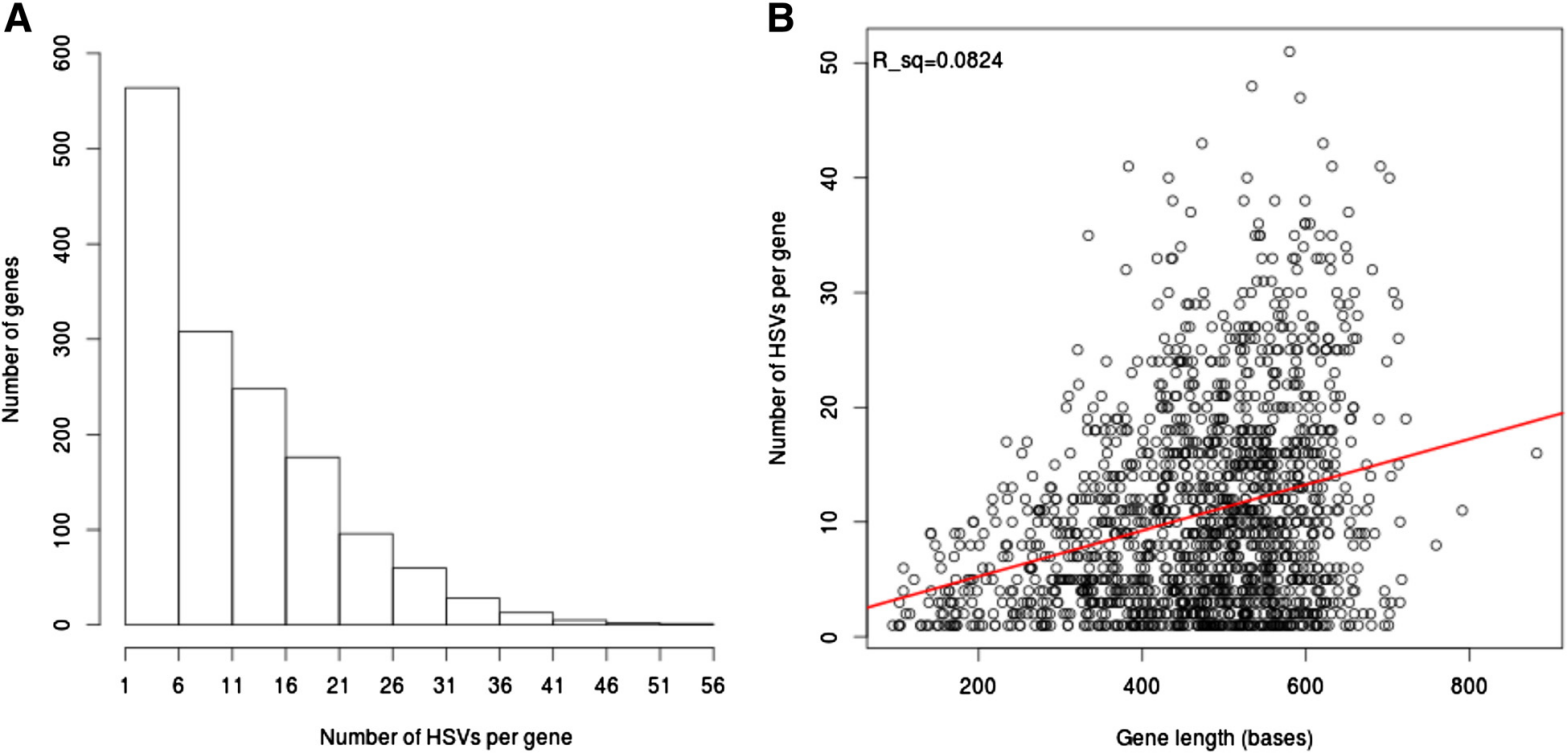
**Distribution of variants for wheat genes on chromosome groups 1 and 5.** HSVs are homoeologue-specific variants detected by nullitetra analysis of wheat RNA-Seq reads. 1,501/2,314 (65%) genes on chromosome groups 1 and 5 have at least 1 HSV expressed in shoots and/or roots. **(A)** Distribution of the number of HSVs per gene for genes with at least 1 HSV. **(B)** Correlation between gene length and number of HSVs, with linear regression fit (red line). The Pearson correlation coefficient r = 0.287 (R squared = .083, *p* < .001).

Genes were called as expressed if they had at least one quality filtered mapped read in at least one euploid wheat sample and at least one nullitetra line. Most genes in the reference transcriptome were expressed, both for group 1 chromosomes: 1,096/1,114 (98%) genes, and group 5 chromosomes: 1,218/1,240 (98%) genes (Additional file 5: Figure S3). The majority of such expressed genes on chromosome group 1 (689/1,096 or 63% genes) and group 5 (812/1,218 or 67% genes) carry one or more HSVs (Table 1) identified in the sequence reads from shoot and/or root tissues via nullitetra analysis. Overall, there were 15 HSVs per kilobase of reference transcriptome, with an average number of HSVs per gene of 10.6 ± 8.8 (Figure 2A). There was only a very weak correlation between gene length and the number of HSVs (R^2^ = .083; Figure 2B). Most variant sites were two-allele HSVs, although we did observe a low frequency (~1%) of sites with 3 homoeologue-specific alleles (Table 1). This lower frequency of three-allele versus two-allele HSVs is expected because three-allele HSVs require two independent (rather than a single) mutations at the same site for their generation. Intriguingly, we identified a significant deficit of D-diagnostic compared with A- or B- diagnostic HSVs (chi-square, *p* < .001) for genes on groups 1 and 5. This deficit is more pronounced for group 1 compared with group 5 genes (Table 1), though the reason for this difference is unclear. In addition, we observed an enrichment of B-diagnostic HSVs on chromosome 1, but not on chromosome 5. The reasons for these deviations in relative frequencies of homoeolocus-specific variants between the two chromosome groups are unknown.

We next combined HSVs co-locating within a region of gene sequence less than the 51 base pair length of an individual sequence read to produce haplotypes. Figure 1B illustrates this process for *BE497595*, a gene with 24 HSVs distinguishing three homoeoloci on chromosomes 5A, 5B and 5D. The boxed region shows three homoeologue-specific haplotypes defined by two A-diagnostic HSVs and a single B-diagnostic HSV. Hence, three homoeolocus-specific haplotypes can be distinguished, despite the absence either of individual 3-allele HSVs or 2-allele diagnostic HSVs for all three homoeoloci. The homoeolocus-specificity of each haplotype is confirmed by its absence in the nullitetra line lacking the respective homoeologous chromosome and by its presence in all other samples (Figure 1C), allowing estimation of the number of homoeoloci of an individual gene that are detectably expressed (e.g., exactly three A, B and D homoeoloci of gene *BE497595*; Figure 1D). For the majority of sequences, haplotypes in the diploids are identical to the corresponding homoeologous haplotypes in euploid wheat (Figure 1C).

**Figure 3:**
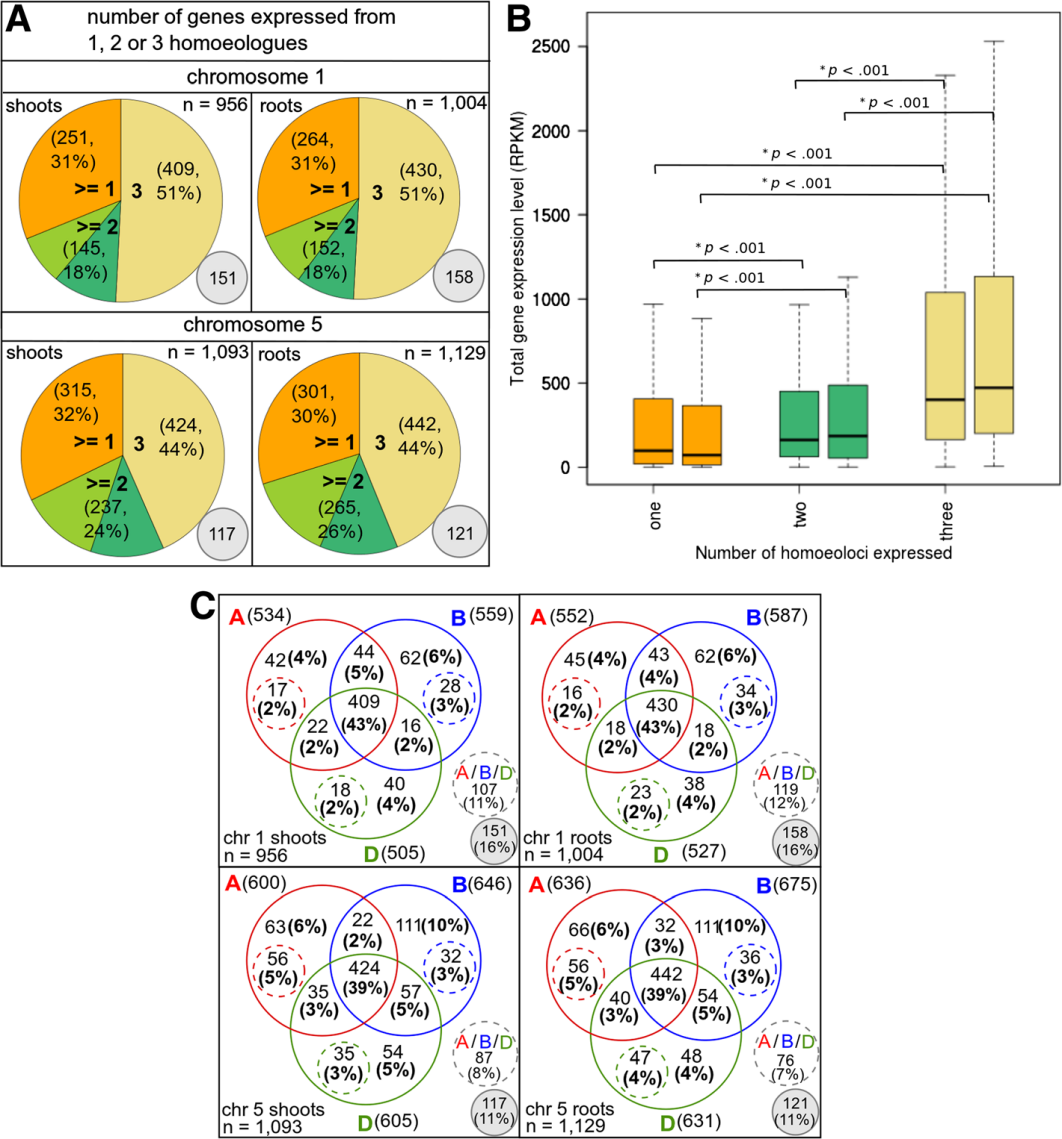
**Expression of up to three homoeoloci for wheat genes on chromosomes 1 and 5. (A)** Genes may be expressed from one, two or three homoeologous chromosomes of group 1 or 5, inferred using nullitetra analysis of RNA-Seq data to detect homoeologue-specific variants (HSVs) or haplotypes in shoots or roots. Genes expressed from 3 homoeologous chromosomes (yellow) have diagnostic sequences for all 3 homoeoloci (A, B and D). Genes expressed from at least two homoeologous chromosomes either have diagnostic sequences for a single homoeolocus, leaving the identity of the second expressed homoeolocus uncertain (light green) or diagnostic sequences for two homoeoloci (dark green). Genes expressed from at least one homoeolocus (orange) have no diagnostic sequences. Genes without evidence for expression from all three homoeoloci are classified as expressed from at least one (≥1) or at least two (≥2) homoeoloci, since they may also be expressed from additional homoeoloci with identical sequences, which cannot be identified. **(B)** Total expression level (RPKM) from all expressed homoeoloci for group 1 and 5 genes expressed from one (orange), two (green) or three (yellow) homoeoloci in shoots (left hand boxes) or roots (right hand boxes). P values are given for a Mann–Whitney test and significant values indicated with an asterisk. **(C)** The number of group 1 (upper panel) or group 5 (lower panel) genes expressed from each combination of homoeologous chromosomes in shoots (left panel) or roots (right panel). Coloured dashed circles indicate genes expressed from the corresponding homoeologous chromosome plus at least one other chromosome whose identity cannot be distinguished. Grey dashed circles indicate genes expressed from a single homoeolocus (A, B or D), whose identity cannot be distinguished. Grey filled circles represent ‘unassigned’ genes whose expressed homoeoloci remained uncertain (see Additional file 15: Table S6).

To assess the qualitative patterns of expression of wheat homoeoloci on chromosome groups 1 and 5, we looked at genes expressed in both biological replicates of each tissue. There were 1,023 (1,058) such group 1 genes expressed in shoots (roots) and 1,164 (1,181) such group 5 genes expressed in shoots (roots). Systematic analysis of homoeolocus-specific sequences allowed us to assign reads, i.e., expression, to specific homoeologous chromosomes. The vast majority of these genes were expressed from the same homoeologues in the two replicates and were selected for further analysis: (956/1,023 (1,004/1,058) group 1 genes expressed in shoots (roots) and 1,093/1,164 (1,129/1,181) group 5 genes expressed in shoots (roots); see Figure 3A). We discovered that at least 45% of such group 1 and group 5 genes whose expression was assigned to homoeoloci are unequivocally expressed from all three possible homoeoloci (833/1,781 genes (47%) in shoots and 872/1,854 genes (47%) in roots; Figure 3A). This figure is an underestimate because expressed homoeoloci are detectable only if expressed at sufficient levels and because homoeologue-specific sequences are required to distinguish homoeolocus expression. Genes expressed from all three group 1 or 5 homoeoloci have a significantly higher total expression level than genes expressed from only one or two homoeoloci, in either shoots or roots (Figure 3B). This is not surprising since genes expressed at high levels were preferentially retained and expressed from multiple copies after polyploidization in other species, including maize [35] and soybean [22]. However, it is difficult to confirm a similar effect in bread wheat, since genes expressed at higher levels will also have a higher probability for detection of all three homoeoloci by nullitetra analysis of homoeolocus-specific sequences in the RNA-Seq data.

### Genes expressed from all three homoeoloci are predominantly expressed from a single homoeolocus

Having established that at least 45% of genes are expressed from all three A, B and D homoeoloci, we next determined the relative contribution of transcripts from each homoeolocus to total gene expression, to test the ‘A = B = D’ (1:1:1) null hypothesis. While we could not be certain that each homoeolocus exists in single copy on each homoeologous chromosome, we could assess the relative contribution from each homoeologous subgenome. To do this we aligned reads using novoalign [64] to a degenerative reference sequence (shown in grey in Figure 1B). For sequence regions with exactly three homoeologue-specific haplotypes, for example those marked by arrows in Figure 1B, reads with A, B and D sequences were quantified and compared using chi-square to a 1:1:1 expectation. Regions with more than three haplotypes were ignored as they most likely contained additional sequence alignments from paralogous genes. Over 93% of the haplotyped regions from all genes showed only two or three homoeologue-specific haplotypes (see Additional file 6: Figure S4), therefore extra sequence alignments from related genes did not significantly constrain our analysis.

**Figure 4:**
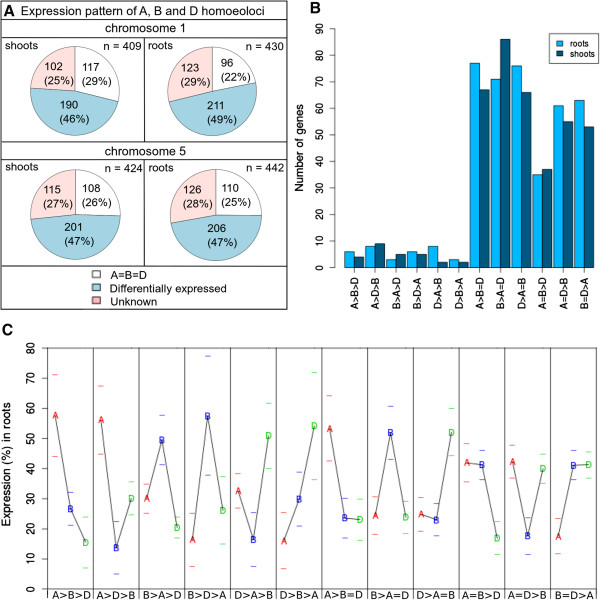
**Biased patterns of expression for homoeoloci on wheat group 1 and 5 chromosomes. (A)** The number of genes expressed from all three homoeoloci in euploid wheat showing either equal expression (‘A = B = D’), a consistent and significant pattern of differential expression bias (e.g., ‘B > A = D’) or classified as unknown (i.e., lack of consistent pattern of significant expression bias) for genes on chromosome groups 1 and 5, in shoot and root tissues. **(B)** The number of genes in roots (light blue) and shoots (dark blue) showing each pattern of differential homoeolocus expression. **(C)** The mean percentage of total transcripts arising from A, B and D homoeoloci for 12 distinct patterns of differential homoeolocus expression in roots (for shoots see Additional file 8: Figure S6). Error bars (-) are given at one standard deviation above and below the mean.

Genes expressed from all three homoeoloci in both biological replicates of a given tissue were classified into those not diverging from the ‘A = B = D’ null hypothesis versus those exhibiting differential expression (Figure 4A). A minority of group 1 and 5 genes showed no sequence regions with evidence of differential expression at *p* < .01 and were classified as equally expressed from all three homoeoloci (‘A = B = D’). This group included 225/833 genes (27%) in shoots and 206/872 genes (24%) in roots (Figure 4A). This estimate of ~1/4 of genes showing ‘A = B = D’ is almost certainly an upper bound, since the statistical power to detect differential expression is governed by the read depth, strength of differential expression and number of sequence differences between homoeoloci. Genes displaying the ‘A = B = D’ pattern have reduced probability for detection of differential expression (see Additional file 7: Figure S5A), due to reduced total read coverage or RPKM (reads per kb per million aligned reads) compared to differentially expressed genes (*p* < .001) and a lower per-site density of HSVs (*p* < .001).

Most genes showed evidence for differential expression of homoeoloci (608/833 genes (73%) in shoots, 666/872 genes (76%) in roots; Figure 4A). These included all genes with quantified regions passing the *p* < .01 threshold. Those passing a Bonferroni-corrected threshold of *p* < .05 were called as significantly differentially expressed, while the remainder were only “suggestive” of differential expression and classified as “unknown” (Figure 4A). We were interested in the diversity and frequency spectrum of different homoeologous expression patterns for genes with significant differential expression. Differentially expressed genes were therefore sorted into all 12 possible categories (Figure 4B): 6 patterns with three distinct levels of expression from each of the three homoeoloci, for example ‘A > B > D’) and a further 6 patterns with two distinct levels of expression, for example ‘A > B = D’. Each pattern with three distinct expression levels ranks the expression of each homoeolocus and is ‘consistent’ with two patterns with just two different expression levels (Additional file 8: Figure S6). For example, ‘A > B > D’ is consistent with ‘A > B = D’ and ‘A = B > D.’ We used a conservative approach to classify genes into each of these categories, requiring the same or a consistent pattern of differential expression in two biological replicates from the same tissue and a consistent pattern across sequence regions (see Methods). For such genes, we combined the two replicates for analysis of the significance of differential expression in each tissue (see Additional file 9: Table S3). For example, in Figure 1D, *BE497595* showed a ‘B > A = D’ pattern in roots for both replicates in both illustrated regions with three homoeolocus-specific haplotypes. This pattern was significant at *p* < .001, with both replicates combined or individually (Additional file 9: Table S3). Around half of genes expressed from all three group 1 or group 5 homoeoloci showed such ‘consistently biased’ patterns of differential homoeolocus expression in shoots (391/833 or 47% genes) and in roots (417/872 or 48% genes). The remainder were added to the “unknown” group of genes, which totalled 217/833 or 26% genes in shoots and 249/872 or 29% genes in roots (Figure 4A). We observed highly similar homoeolocus expression patterns from the two replicates of each tissue, with less than 7% of ‘unknown’ cases in either shoots (14/217 genes) or roots (15/249 genes) caused by conflicting patterns of expression. Note that while we do not focus on the expression of homoeoloci in the nullitetras here, Figure 1C shows that for *BE497595*, all the nullitetras except for N5BT5A show an expression pattern consistent with a dosage response to the extra copies of the ‘tetra’ genome. N5BT5A shows the opposite response, a negative dosage compensation in which the contribution from the ‘tetra’ genome (A) is lower than that of the other ‘diploid’ genome (D).

All twelve patterns of differential homoeolocus expression occur in both shoots and roots, though not with equal frequency (Figure 4B; Additional file 8: Figure S6). Less than 10% of differentially expressed genes had three distinct expression levels (e.g., ‘A > B > D’) in shoots (27/391 genes) or roots (34/417 genes). This is an underestimate since these genes are expressed at significantly higher levels compared with genes having two distinct expression levels (*p* < .001, Additional file 7: Figure S5C), which improves the statistical power to detect differential expression of homoeoloci. Cases where one homoeolocus dominates expression (e.g., ‘A > B = D’), were significantly more frequent than cases with co-upregulation of two homoeoloci (e.g., ‘A = B > D’) (Figure 4B; chi-square, *p* < .001 in shoot and roots). On average, among such cases, the predominant homoeolocus contributes ~55% of total expression, while the other two homoeoloci play a subsidiary role and supply the remaining 45% (Figure 4C; see Additional file 8: Figure S6). A small number of genes exhibit extremely strong homoeolocus expression bias. For example, the group 5 gene *BF484913* encoding a predicted enolase shows ‘B > A = D’ in shoots, with an average of 86 ± 5% expression arising from the B homoeolocus (see Additional file 8: Figure S6J). However, the extent of expression bias shows no clear relationship with the total gene expression level from all three homoeoloci (see Additional file 10: Figure S7).

There is no global bias for preferential expression of homoeoloci from a particular A, B or D homoeologous subgenome (Figure 4B). In contrast, when two of the three expressed homoeoloci dominate total gene expression, there is a striking difference in the frequencies of the three possible co-upregulated pairs (Figure 4B), with A and B homoeoloci significantly underrepresented compared with ‘A, D’ or ‘B, D’ combinations (chi-square, *p* < .001 in shoots and in roots). However, we do not observe a similar deficit for the alternative pattern in which A and B homoeoloci are expressed at the same level (‘D > A = B’) compared with either ‘B > A = D’ or ‘A > B = D’.

To explore the factors underlying differential homoeologous expression patterns in euploid wheat, we looked at the possible role played by biological function. We used the FatiGO functional analysis tool [66] to explore the relationship between biological function and homoeologous gene expression patterns. We found that genes expressed from all three homoeoloci in either shoots or roots were significantly enriched for only a single term, cellular component GO:0005739 (mitochondria), compared with genes expressed from only one or two homoeoloci (Fisher exact test, *p* < .001). However, we did not find any functional trends (significant GO terms) to explain specific patterns of expression of homoeoloci (e.g., ‘A > B = D’).

We experimentally validated, using two independent approaches, our computationally determined homoeologue-specific variants (HSVs), and the differential homoeolocus expression patterns observed in roots, for six genes expressed from all three A, B and D homoeoloci. First, semi-quantitative RT-PCR and second, PCR product cloning into a bacterial vector followed by Sanger sequencing, using the same root RNA samples used for RNA-sequencing (Additional file 11: Figure S8 and Additional file 12: Table S4). All HSVs were validated for these six genes, giving a zero rate of false positives. Some HSVs in the amplified sequences were missed by our nullitetra analysis strategy, with an average false negative rate of 18%. HSVs were missed for various reasons, including insufficient coverage across all nullitetra samples in the region of the HSV, absence of homoeologue-specificity in one or more nullitetras, or sequence divergence between Chinese Spring and EST sequences, which originated from a variety of *Triticum* species (Additional file 2: Table S2). All differential expression patterns were validated for each gene using one or both of these methods. Therefore, our strategy can successfully determine the homoeolocus expression patterns of wheat genes using fragments of gene sequence, rather than full gene models.

### Similar patterns of differential homoeologous expression in two wheat tissues

We next determined the degree to which expression patterns for genes expressed from all three A, B and D homoeoloci are shared between shoot and root organs of the wheat plant, based on 787 genes expressed from all three group 1 or 5 homoeoloci in both tissues. A comparison of homoeolocus expression patterns was possible despite significantly higher average total expression levels in roots compared with shoots, of unknown cause (*p* = .014; Additional file 13: Figure S9). Of the above 787 genes, 373 displayed either significant differential expression or equal expression of all three homoeoloci (‘A = B = D’) from both tissues. 218/373 (58%) of these genes showed agreement, defined where both tissues showed either the same pattern of expression, or two ‘consistent’ patterns of differential expression (see Additional file 8: Figure S6). Most disagreements (121/155 genes) were cases where the three homoeoloci were differentially expressed in one tissue, but equally expressed in the other (‘A = B = D’). For most such cases (77/121 or 64% genes), the tissue showing equal expression also had lower coverage, suggesting that some of these disagreements may be false positives caused by the statistical difficulty in confirming differential expression of homoeoloci for genes expressed at low levels. Of more interest are those 34 genes with conflicting patterns of differential homoeolocus expression (see Additional file 14: Table S5). For example, *BE606302* is a highly expressed uncharacterized protein similar to the *Arabidopsis* gene *TET3*, which shows a ‘B > A = D’ pattern in shoots but has a significantly higher contribution from the A homoeolocus in roots, showing ‘A = B > D’ (35% ± 1% of expression from the A homoeolocus in roots versus 24 ± 2% in shoots). The biological relevance of these tissue-specific homoeolocus expression patterns warrants further investigation.

### Detecting the presence and expression of homoeoloci

The remaining ~55% of wheat genes on chromosome groups 1 and 5 were not detectably expressed from all three possible homoeoloci (948/1,781 genes in shoots and 982/1,854 genes in roots; Figure 3A). Such expression patterns may be generated by physical loss (deletion) of homoeoloci, absence of transcripts from a particular homoeolocus (due to epigenetic transcriptional silencing or transcript-null mutation), and includes genes without diagnostic homoeolocus-specific sequences. Distinguishing these causes is a challenge because lack of evidence for presence or expression of a homoeolocus on a particular homoeologous chromosome may not mean it is absent or not expressed. Nevertheless, we proceeded to categorize the presence and expression of each gene based on several sources of evidence (see Additional file 15: Table S6).

To infer presence of a gene on a particular homoeologous chromosome, we focused on group 1 chromosomes and combined three sources of evidence. First, the deletion bin mapping dataset of Qi et al. [58,63], which assigned genes to homoeologous chromosomes according to the presence or absence of a restriction fragment in Southern blots from a series of aneuploid and deletion wheat lines [58,63]. Second, nullitetra analysis of our shoot and root RNA-Seq data from euploid wheat and its derived nullitetra lines to identify transcribed homoeologue-specific sequences (individual HSV sites or haplotypes). Third, the group 1 homoeologous chromosome specific DNA sequence assemblies of Wicker et al. [52], generated by sequencing flow sorted chromosomes from double ditelosomic lines of Chinese Spring wheat (Additional file 16: Table S7). This dataset included assemblies from the short and long arms of chromosomes 1A and 1B, and from the long arm of 1D, but lacked assembled sequences from the 1D short arm due to chromosomal rearrangement in the corresponding wheat line. We identified matches for 642 (58%) of our 1,114 group 1 genes using BLAST comparison with our homoeologue-specific sequences. The absence of sequences from chromosome 1DS [52] means we have most likely underestimated the number of genes present on the 1D homoeologue.

**Figure 5:**
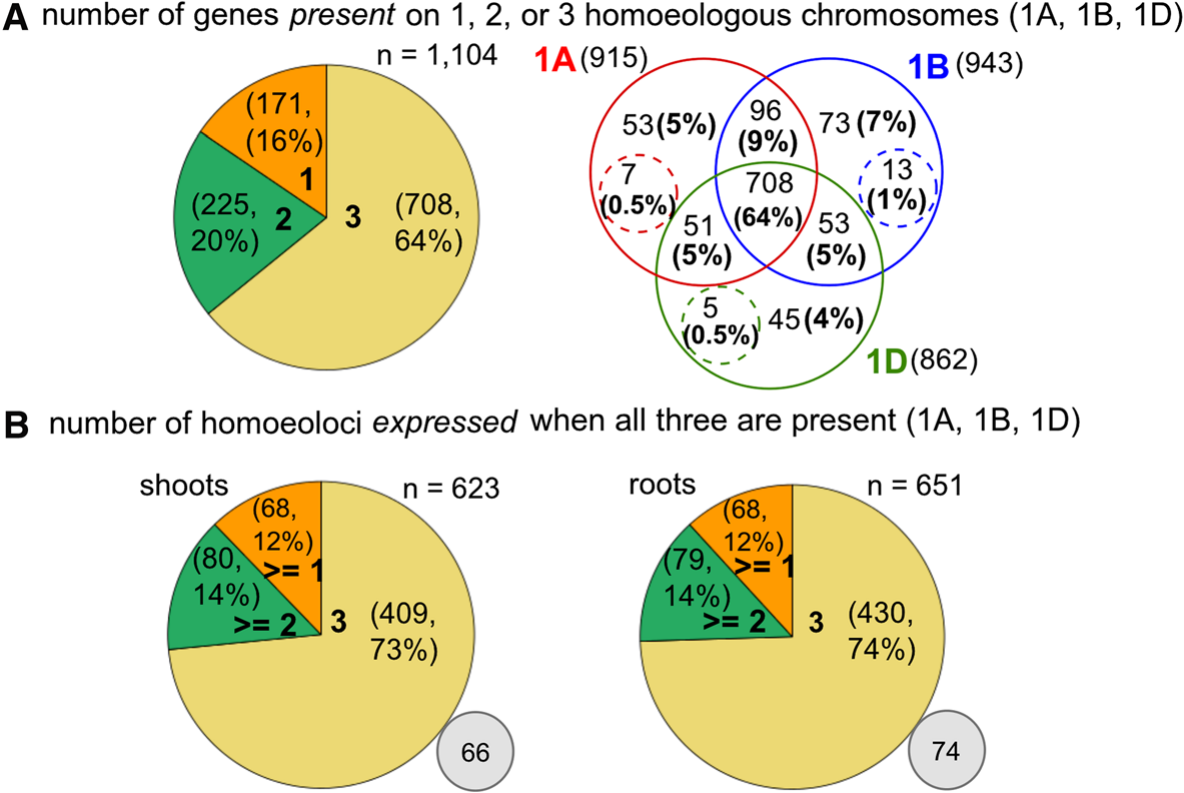
**Presence and expression of up to three homoeoloci for wheat genes on group 1 chromosomes. (A)** Presence of a gene on 1A, 1B, and 1D homoeologous chromosomes is inferred using nullitetra analysis of RNA-Seq data combined with deletion bin mapping data [58,63] and chromosome 1 arm-specific assemblies from Wicker et al. [52]. The pie chart shows the number of genes present on one, two or three homoeologous chromosomes, while the Venn diagram shows the number of genes present on each combination of chromosomes. Dashed circles indicate genes present on the corresponding homoeologous chromosome plus at least one other chromosome whose identity cannot be distinguished. **(B)** Genes with evidence for presence on all three homoeologous chromosomes of group 1 are expressed from at least one, at least two, or all three homoeoloci in shoots (left panel) and roots (right panel) according to the presence of group 1 homoeologue-specific sequences. Grey circles show the number of genes with expression classified as ‘unassigned’ (see also Additional file 15: Table S6).

We discovered evidence for substantial gene loss during the evolutionary history of bread wheat (Figure 5A). Among 1,104 group 1 genes, (excluding 10 group 1 genes with inconsistent expression of homoeoloci between replicates), we found 708 (64%) genes present in three copies (1A, 1B and 1D; Figure 5A). There are more genes on chromosome 1B (943 genes) than on 1A (915 genes), which in turn exceeds the number present on 1D (862 genes) (Figure 5A). We estimate that most (at least 60%) homoeolocus loss events occurred after the two polyploidization events that created bread wheat. For example, there are 44 genes present on chromosomes 1B and 1D (and expressed from one or both), but absent from 1A, 25 (57%) of which are detectably expressed (with at least one quality filtered read) in the A genome diploid. Similarly, 73 genes are present on 1A and 1B but absent from 1D, of which 47 (64%) are expressed in the D genome diploid.

### Widespread homoeolocus loss and silencing mean ~1/3 wheat genes are expressed from only a single homoeolocus

For the majority of genes from chromosomes 1 and 5, we confirmed the identity of all expressed homoeoloci in both shoots (635/956 (66%) group 1 genes; 766/1,093 (70%) group 5 genes) and roots (654/1,004 (65%) group 1 genes; 793/1,129 (70%) group 5 genes), as indicated by the solid circles in Figure 3C; see also Additional file 15: Table S6. For the remaining genes, the identity of one or more homoeoloci was uncertain, as indicated by dashed circles in Figure 3C. For example, genes with diagnostic sequences (HSVs) for a single homoeologous chromosome (e.g., 1A) must be expressed from this chromosome, plus at least one other chromosome whose identity cannot be confirmed. Genes present on more than one homoeologous chromosome and with no HSVs are likely to be expressed from a single indistinguishable homoeologue (e.g., 1A or 1B homoeologues for genes present on 1A and 1B chromosomes). We found that 1,772/1,979 (90%) of group 1 and 5 genes showed evidence for expression from the same homoeoloci in both shoots and roots, although a small number showed evidence for organ-specific transcript absence for one or more homoeoloci; Additional file 17: Table S8).

We observe frequent loss of expression (transcript absence) of group 1 homoeoloci (Figure 3A). For example, among genes with evidence for the presence of three distinct homoeoloci, 12% appear to be expressed from only a single homoeolocus in both shoots and roots, while another 14% are expressed from only two of the three homoeoloci (Figure 5B). Overall, around one third of wheat genes are expressed from just one homoeolocus, having lost or silenced the other two copies (566/1,781 (32%) genes in shoots and 565/1,854 (30%) genes in roots; Figure 3A). If homoeoloci are lost or lose transcription function randomly, genes expressed from two homoeoloci should be more frequent than those expressed from one. Intriguingly, however, we found that genes on group 1 are significantly more likely to be expressed from just one homoeolocus than from two homoeoloci in shoots (251 genes versus 145 genes, chi-square, *p* < .001; Figure 3A) and in roots (264 genes versus 152 genes, *p* < .001; Figure 3A). The same is true for group 5 genes expressed in shoots (315 genes versus 237 genes, *p* < .001), though the difference is not significant at cutoff *p* < .05 for roots (*p* = .083). We conclude that in terms of its gene expression, the bread wheat genome is tending towards diploidization in two ways; first, we estimated that only ~64% of group 1 genes are present in three homoeologous copies, indicating substantial gene loss has occurred (Figure 5A). Second, while there is still more than one homoeologous copy for at least 84% of group 1 genes (Figure 5A), only 69% of these genes are expressed from more than one copy in either shoot or root tissues (Figure 3A), which could indicate substantial homoeolocus silencing.

Assuming we have correctly identified all present and expressed homoeoloci, we found a comparable frequency of genes showing physical absence (loss) and transcript absence (silencing) of homoeoloci. In the absence of evidence for expression from a particular homoeologous chromosome, we identified cases of potential homoeolocus loss where there was no evidence for its presence, or of transcript absence where there was evidence for its presence. Among 805 genes whose expression in shoots was assigned to one or more group 1 homoeologous chromosomes, 211 (26%) have lost one or two homoeoloci, while 193 (24%), have silenced one or two homoeoloci (Additional file 15: Table S6). Similarly, for genes expressed in roots, 227/846 (27%) have lost one or two homoeoloci, while 201/846 (24%) have silenced one or two homoeoloci. We conclude that physical loss and transcript absence have both played an important role in the loss of homoeologous gene expression in hexaploid bread wheat.

### B-subgenome homoeoloci contribute disproportionately to wheat gene expression

B subgenome homoeoloci not only exceed A or D homoeoloci in number but also contribute more to the bread wheat transcriptome (Figure 3C). Significantly more genes are expressed from B homoeoloci of groups 1 or 5 than from either A or D homoeoloci, both in shoots (A(1,134), B(1,205), D(1,110), chi-square, *p* = .046) and in roots (A(1,188), B(1,262), D(1,158), *p* = .036). Among genes expressed from a single homoeolocus, significantly more are expressed from B (6-10% genes) than from either A or D chromosomes (4-6% genes), both in shoots (Figure 3C, chi-square, *p* = .013 for group 1; *p* < .001 for group 5) and in roots (Figure 3C, chi-square, *p* = .016 for group 1; *p* < .001 for group 5). It is possible that there are more genes detectably expressed from 1B and 5B homoeologous chromosomes than from A or D chromosomes due to a higher frequency of B homoeolocus-specific variants. Indeed, it is plausible that B homoeologous chromosomes are more polymorphic than A or D chromosomes, given the differences in both mating systems and genome ages of the parental diploid species [63,67,68]; the B homoeologous genome was acquired from a cross-pollinating species related to *Aegilops speltoides*, while the A and D homoeologous chromosomes trace their origins to self-pollinating diploids [67,68]. However, this higher frequency of B-specific HSVs is not common to our entire dataset (not seen on chromosome 5 for example, see Table 1). We therefore conclude that B-genome homoeoloci tend to contribute more to wheat gene expression than do A or D genome homoeoloci.

A natural and long-standing question is whether A, B and D homoeoloci are equally likely to be transcript-absent [61,69-71]. It is possible that genes from particular wheat homoeologues have been preferentially silenced during polyploid evolution. Contradictory evidence exists for preferential homoeologue silencing in wheat, with one study showing D genome homoeoloci to be silenced twice as frequently as A or B homoeoloci [72], and others showing weak evidence for preferential silencing of D homoeoloci [70], or indeed no preferential silencing at all [61,69]. Here we focussed on genes with evidence for presence on all three group 1 homoeoloci, but detectable expression for only two homoeoloci (see Additional file 15: Table S6). In shoots, there are more genes with a transcript-absent D homoeolocus (23 genes) compared to transcript-absent B (17 genes) or A (11 genes) homoeoloci, though the difference was not significant. In roots, there were significantly more genes with a transcript-absent D homoeolocus (21 genes) than genes with a transcript-absent B (11 genes) or A (12 genes) homoeolocus (chi-square, *p* = .043). Most of the transcript-absent A homoeoloci (10/12) were expressed in the A genome diploid. Similarly, most transcript-absent D homoeoloci (17/21) were expressed in the D genome diploid. While our results corroborate previous work demonstrating preferential post-polyploidization transcript absence for homoeoloci on the D genome [70,72], it is nonetheless particularly unexpected given that the D genome was integrated into hexaploid wheat only ~8,500 years ago. The mechanism(s) and reversibility of such transcriptional silencing are not well understood, and may involve methylation [14], mutation in the promoter region, or other gene silencing mechanisms.

## Discussion

Understanding the structure and function of the bread wheat genome is a major challenge, due to its complex evolutionary history of successive hybridization and allopolyploidization. In particular, it has remained unclear on a large scale whether the three ancestral diploid genomes contribute equally or differentially to wheat gene expression, and hence to wheat phenotypes. Our study explores this question in detail, using an extensive, systematic approach for dissecting the relative contributions of the three potential homoeoloci (A, B and D) to total wheat gene expression.

Several studies on the differential expression of homoeoloci in wheat have focussed on the expression of a relatively small number of genes [46-48,61,71]. Subsequent genome-wide assessments have so far used microarrays, which are difficult to interpret for the expression of closely related genes [42-45]. Such studies have therefore been unable to separate the contributions of individual homoeoloci, particularly the A and B homoeoloci originating from the parental tetraploid species. Here we have developed a novel analysis of euploid bread wheat and its derived nullisomic-tetrasomic lines bearing specific chromosome substitutions. Using high coverage RNA-sequencing, we have successfully distinguished gene transcripts from all three hexaploid wheat homoeoloci (A, B and D). This direct approach alleviates the issue of chimeric sequence assembly encountered in previous work [40,55], for genes on those chromosome groups for which nullitetra sequences are available (groups 1 and 5 in the present study). This allowed direct quantification of gene transcripts for which homoeolocus specificity was established.

Combining our RNA-Seq data with published deletion bin mapping data [58,63] and chromosome arm-specific assemblies of group 1 chromosomes [52], we have assessed the physical distribution and expression of a sample of 2,314 wheat genes present on groups 1 and 5 of the seven homoeologous bread wheat chromosome groups. We estimate that at least 45% of genes are expressed as three distinct homoeoloci (A, B and D) in both shoot and root tissues, as would be expected based on the hexaploid nature of the wheat genome. The majority of such genes display a biased pattern whereby the output of a single homoeolocus dominates total gene expression. Similar studies of other allopolyploids including oilseed rape [73], and cotton [28,32,74-76], have also shown that genomically biased expression is common across a range of vegetative and floral tissues. In addition, all possible patterns of biased homoeolocus expression occur from various genes and tissues, consistent with our results, which show all twelve possible patterns of differential expression from three wheat homoeoloci. Previous work on polyploid wheat [61] and cotton [76], and on diploid *Arabidopsis*[77], has shown frequent cell type-specific homoeolocus (or duplicate gene) expression patterns. Perhaps surprisingly, we uncovered limited evidence for contrasting patterns of differential expression between homoeoloci in two wheat tissues. Extending our work to the expression analysis of specific cell-types, developmental stages or growth environments would likely show more variation in the expression of homoeoloci. Our analysis strategy involves simultaneously identifying homoeologue-specific variants and quantifying their contribution to the transcriptome. As such, it has focused on genes that are highly expressed and highly polymorphic. It is possible that the expression of homoeoloci is more variable for such genes. As the international wheat community continues to make rapid progress towards completion of an annotated reference wheat genome sequence [40,78], we will in the future be able to assess the generality of the patterns we have observed here for all wheat genes, including those expressed at low levels and those with subtle or highly skewed expression of homoeoloci.

Genes expressed from all three homoeoloci in either tissue were enriched for the mitochondrion cellular component GO term. This supports a key role for mitochondria in providing hexaploid wheat with more efficient energy-generating mechanisms than its diploid and tetraploid relatives [79]. However, there were no functional trends (significant GO terms) to explain specific patterns of expression of homoeoloci (e.g., ‘A > B = D’). This may be due to insufficient statistical power, but it is also consistent with recent work [45] showing that while the gene expression pattern itself is heritable, the identity of genes showing various patterns can be highly stochastic and involve diverse gene functions. We did not find any global bias towards preferential expression of homoeoloci from a particular homoeologous subgenome, for those genes expressed from all three homoeoloci. In contrast, bias in favour of D genome homoeologues has been reported in five cotton species [33,34]. In addition, the transcriptomes of two resynthesized hexaploid wheat lines contained more genes showing tetraploid parental dominance than diploid parental dominance [45], though the contributions of A and B homoeoloci could not be separated. Observations in other polyploid species, including cotton [32,76], oilseed rape [73] and *Spartina*[80], suggest there are both immediate and long term alterations in homoeologous gene expression patterns after polyploidization. We propose that the biased patterns of homoeolocus expression we observed are attributable to coordinated (as opposed to independent) divergence in the regulation of individual homoeolocus expression levels following polyploidization. This possibility is strengthened by our observation that in cases where two homoeoloci equally dominate total gene expression, dominance of A and B homoeoloci is less likely than dominance of A and D or B and D homoeoloci. This may reflect the fact that A and B genome homoeoloci have been associated in bread wheat (and progenitors) for ~0.5 million years, whilst D genome homoeoloci were only incorporated ~8,500 years ago.

We estimate that around 55% of wheat genes are no longer expressed from all three potential A, B and D homoeoloci, due to both extensive homoeolocus loss and extensive transcript-absence of homoeoloci. Homoeolocus loss has been extensive since only 64% of genes exist in A, B and D copies. This result is consistent with wheat whole-genome sequencing [40], which showed most wheat genes with orthologous copies in related cereals have between one and five copies, with a peak of two copies. In addition, assembly of sequences from wheat chromosomes 7A, 7B and 7D showed that 54% of genes remain present on all three homoeologues [81]. We also observed more B homoeoloci compared with A or D homoeoloci, supporting earlier observations based on bin mapping data [58,63]. This is likely to reflect, in part, the accumulation of non-syntenic genes on the 1B homoeologue [52], as well as differential loss of homoeoloci from particular homoeologous chromosomes, which is commonly observed in paleopolyploid species [17-22]. This enrichment for B homoeoloci was not detected in the genome-wide analysis of wheat gene assemblies [40], perhaps due to poor discrimination of B genome homoeoloci. A different pattern has been observed for genes on group 7 chromosomes, with homoeolocus loss greater from chromosomes 7A and 7B than from chromosome 7D [81], which was attributed to two rounds of gene fractionation following polyploidization during the evolutionary history of bread wheat. The causes of these differences are unknown and might involve chromosome group-specific patterns of homoeolocus presence/loss.

In both shoot and root tissues, an unexpectedly high proportion (~1/3) of all genes appear to be expressed solely from a single homoeolocus and we have no reason to believe this does not apply to all wheat tissues and to genes on chromosome groups other than 1 or 5. Among genes present in three copies (1A, 1B and 1D), 26% have silenced one or more homoeoloci, which agrees well with a previous estimate of 29% of genes in the established hexaploid [70]. In a study of synthetic wheat, only 5% of genes showed evidence for homoeolocus silencing, suggesting that wheat homoeoloci are silenced gradually. Overall, our results show that the bread wheat genome has undergone extensive “diploidization” [5]. This is a particularly interesting finding in the light of other observations. For example, hexaploid wheat can tolerate at least a ten times higher mutation rate (mutated base pairs per kb of sequence) than diploid wheat [82], which has made hexaploid wheat particularly well suited for TILLING (targeting induced lesions in genomes). This is usually explained by the redundancy provided by homoeoloci in the hexaploid genome [82,83]. The fact that nullisomic-tetrasomic lines of wheat can be produced exhibiting only minor phenotypic effects [58,83] further testifies to the buffering effects of polyploidy. This could be explained at least in part by the reversibility of gene silencing, as demonstrated previously [70], and by the duplication of homoeologues across more than a single chromosomal group.

## Conclusions

The analysis strategy we have developed should be of great use for the assembly of homoeologue-specific sequences in wheat genome and transcriptome sequencing projects. Wheat displays substantial levels of variation at the phenotypic, genetic and epigenetic levels, which is of paramount importance to wheat breeding. Our work gives a comprehensive understanding of the homoeolocus expression patterns characteristic of bread wheat genes, providing a key stepping-stone towards understanding the relationship of wheat variation with differential homoeolocus expression patterns. In addition, the observation that bread wheat is tending towards functional diploidy has important practical implications for the strategies employed by breeders in the development of improved wheat strains.

## Methods

### Plant material

Three spikelets of *T. aestivum*, *T. urartu*, *Ae. speltoides* and *Ae. tauschii* and chromosomes 1 and 5 nullisomic-tetrasomic lines were soaked on Whatman filter paper in Petri dishes. The spikelets were stratified at 4°C for 2 days in the dark before extracting the seeds from the spikelets. The seeds were soaked on Whatman filter paper in Petri dishes and stratified at 4°C for a further 2 days. Three replicates for each plant were grown hydroponically in a controlled environment room (16/8 h light/dark cycle at 21°C). Root and shoot tissue samples were collected when the fifth leaf appeared. At this point, the fourth leaf was taken as the shoot sample.

### Construction of reference transcriptome from wheat EST sequences

Sequences for 6,419 Chinese Spring wheat deletion bin-mapped 5′ ESTs from a broad range of libraries [58,63] were acquired from the website of the wheat EST project (http://wheat.pw.usda.gov/cgi-bin/westsql/map_locus.cgi) and processed to remove polyA tails. These sequences were already assigned to specific homoeologous chromosomes according to the presence or absence of a restriction fragment in Southern blots from a series of aneuploid and deletion wheat lines [58,63]. ESTs present on one or more homoeologues of group 1 (1,123) and group 5 (1,247) chromosomes were assembled into groups of similar, overlapping ESTs (contigs) using CAP3 software [84] to reduce gene redundancy. Using a stringency level of 95% sequence identity over a 40 bp overlap, 17 ESTs on chromosome 1 were assembled into 8 contigs and 14 ESTs on chromosome 5 were assembled into 7 contigs, giving a total of 15 contigs for both chromosome groups. The final set therefore consisted of 2,354 ESTs/contigs (‘genes’), with 1,114 on group 1 chromosomes and 1,240 on group 5 chromosomes. Gene sequences were concatenated and spatially separated with 200 ‘N’ nucleotides to produce a consensus wheat transcriptome with 505,132 and 567,044 bases of genic sequence for chromosomes 1 and 5 respectively. Note that a near-complete assembly of genome-wide wheat gene sequences has subsequently become available [40].

### Functional assignments

Genes were assigned functions using either 1) translated nucleotide query (BLASTx) against the database of all non-redundant proteins or 2) BLASTn against database release 12.0 of TIGR TCs (tentative contigs) for *Triticum aestivum* and extraction of GO assignments of the TCs showing the best hit. All BLAST hits were filtered using e-value 1e^-10^. Extracted GO terms were used in FatiGO within the Babelomics suite [66] for functional over-representation analysis.

### Next generation RNA sequencing data

Total RNA was extracted using the TRIzol Reagent (Invitrogen) from leaf and root tissue of the following wheat samples from the John Innes Centre Wheat Germplasm collection: 1) Sears’ Chinese Spring hexaploid (euploid wheat), 2) the six chromosome 1 compensating nullitetras, 3) the six chromosome 5 compensating nullitetras, 4) D genome diploid (accession code 2220007), and 5) A genome diploid (accession code 1010005). Non-normalized cDNA libraries were created to show the realistic gene expression levels in each of the 32 wheat samples. Each library was paired-end sequenced (mostly 51 bp read length) in at least four lanes of the Illumina Genome Analyzer II (GAII) at the Wellcome Trust Centre for Human Genetics, Oxford (see Additional file 1: Table S1). The average number of sequence reads per wheat sample was 232 million.

### RNA sequencing read mapping

Sequence reads were mapped to our reference transcriptome using three mapping software tools. First, Maq version 0.7.1 [85] was used with default parameter settings to detect homoeologue-specific variants (HSVs) in euploid wheat sequence reads using nullitetra analysis. Two software tools, novoalign V2.07 [64] and gsnap [86], were used for quantification of homoeologous gene expression. These tools are designed to reduce read mapping biases [87], particularly preferential mapping of reads derived from the homoeolocus matching the reference sequence. Novoalign uses a degenerate reference constructed by replacing the allele in the reference sequence with the corresponding genetic ambiguity code (IUPAC) at HSV sites. For example, a ‘T/C’ HSV was encoded with a ‘Y’ in the degenerative reference sequence. Gsnap supplements the reference sequence with a list of all known HSVs and their corresponding homoeoloci. It produced virtually identical results (not reported here) to those from novoalign. All read alignments were converted to the standard format using SAMtools version 0.1.5c [88] and quality filtered using custom Perl scripts (see Additional file 4: Figure S2). Entire reads were excluded based on: 1) non-unique mapping (mapping quality zero), 2) nucleotide(s) inserted or deleted relative to the reference, 3) mapping quality <30. Mapped pairs with an unexpectedly large insert size (>700 bp) were also excluded based on an expected library insert size of 250 ± 50 bp. Within each mapped read, bases with Phred quality score <20 were excluded. For each gene, the relative total expression level from all expressed homoeoloci was calculated from the total number of reads mapping to the gene sequence, normalized by both the sequence length and the total number of mapped reads in the sample, to give a standard RPKM measure of expression (reads per kb per million aligned reads). The Mann–Whitney test was used to compare RPKM between groups of genes due to lack of normality of the RPKM distribution.

### Detection of homoeologue-specific sequences

Custom Perl scripts were used to identify homoeologue-specific variants (HSVs), defined as single base differences between homoeolocus sequences. Candidate HSVs were identified at nucleotide positions (‘sites’) in the reference sequence where 2 or 3 different bases (homoeoloci) were detected in the aligned euploid wheat reads from the combined shoot and root RNA-Seq datasets. Sites with coverage less than 10 mapped reads were excluded. A candidate homoeolocus was defined for bases with either frequency ≥ 2 (and at least 3% of mapped reads) or with frequency ≥ 15 reads. True HSVs that were ‘diagnostic’ (i.e., specific) for a particular homoeologue (A, B or D) were confirmed by the presence of the diagnostic base at a particular site in all nullitetra lines except for those lines lacking the corresponding homoeologous chromosome.

Homoeologous sequence variants (HSVs) co-locating within a region of gene sequence less than the 51 base pair sequence read length were combined to produce haplotypes. Homoeolocus-specificity of each haplotype was confirmed by its absence in the nullitetra line lacking the respective homoeologous chromosome and by its presence in all other samples. Haplotypes with read frequency < 2 were discarded.

### Expression patterns of genes expressed from all three homoeoloci

For genes with homoeolocus-specific sequences from all three homoeoloci, duplicate read pairs with the same sequence, orientation and start position were removed. The chi-square test was used to identify differential expression of homoeoloci. For each sequence region with exactly three homoeolocus-specific haplotypes, and a minimum coverage of 18 reads, the frequency of mapped reads with each haplotype was tested against the null hypothesis of equal expression from all three homoeoloci (1:1:1, ‘A = B = D’). Unadjusted *p* < .01 were considered “suggestive” of differential expression. Such regions were confirmed as “significant” if passing a Bonferroni adjusted threshold of *p* < .05 (accounting for the number of regions tested), based on combined reads from two biological replicates. Sequence regions indicating possible read alignments from paralogous genes were ignored, including those with additional non-homoeolocus-specific haplotypes in euploid wheat, or with more than one haplotype in diploid wheat read alignments. Genes without any “suggestive” evidence of differential expression were assigned an equal expression pattern (‘A = B = D’).

For genes with “significant” differential expression of homoeoloci, we assigned one of twelve patterns, including six with all three homoeoloci expressed at distinct levels (A > B > D, A > D > B, B > A > D, B > D > A, D > A > B, D > B > A) and a further six with two homoeoloci expressed at indistinguishable levels (A > B = D, B > A = D, D > A = B, A = B > D, A = D > B, B = D > A). For each quantified region, the three pairs of haplotypes (‘AB’, ‘AD’ and ‘BD’) were compared to a null hypothesis of equal expression from each homoeolocus (1:1), to determine whether each pair of homoeoloci was expressed at equivalent levels (e.g., ‘A = B’) or distinct levels (e.g., ‘A > B’), using *p* < .01. Genes with evidence for a single pattern of differential expression in both biological replicates were called “consistently biased”. For such genes, the expression level from each homoeolocus was calculated by averaging the proportions of aligned reads with each homoeolocus-specific haplotype from all quantified regions, weighted by the total number of aligned reads in each region. The remaining genes were called “unknown,” and excluded from further analysis. Genes in the “unknown” category included those with evidence for multiple, inconsistent patterns of differential expression, (e.g., both ‘D > A = B’ and ‘A = D > B’) or inconsistent patterns between replicates. This is explained by various factors, including low expression levels (leading to lack of statistical power to distinguish expression of homoeoloci), alternative splicing, or RNA-Seq related biases [86]. We also assigned genes as “unknown” if they showed differential expression only at the “suggestive” level.

### Assigning presence and expression of genes among homoeologous chromosomes

A gene was defined as present on a specific homoeologous chromosome (A, B, or D) in the presence of evidence from deletion bin mapping [58,63] or group 1 chromosome arm-specific assemblies [52]. The existence of homoeolocus-specific i.e., diagnostic sequences (either individual sites (HSVs) or haplotypes) from RNA-Seq enabled us to define both presence on, and expression from, the corresponding homoeologous chromosome (see Additional file 4: Figure S2). BLAST searches were used to find the best match for each group 1 gene to sequences from chromosome arms 1AL, 1BL, 1DL, 1AS and 1BS [52]. No sequences were available for chromosome arm 1DS; see Additional file 16: Table S7. Significant hits were defined using an e-value cut-off of 1e^-10^ and required to have a minimum stretch of 60 identical bases.

### Experimental validation of homoeologue sequence variants (HSVs)

Two micrograms of total RNA extracted from euploid (CS) and nullitetra wheat root samples using the TRIzol Reagent (Invitrogen) was reverse-transcribed to synthesize first-strand cDNA using the SuperScript II First-Strand Synthesis System for RT-PCR Kit (Invitrogen). cDNA was treated with Qiagen RNase A to remove any residual RNA. We selected 10 genes with differential expression of homoeoloci and with three homoeolocus-specific tentative contigs (TCs) identified from a BLAST search of the *T. aestivum* TIGR database (Additional file 12: Table S4A). The homoeologous sequences of each selected gene were extended from the 3′ end using the homoeologous TC sequences to provide highly variable regions for homoeolocus-specific primer design. All PCR primers were designed using Primer 3 (http://frodo.wi.mit.edu/) to amplify either all three homoeoloci (using common forward and reverse primers) or specific homoeoloci (using homoeolocus-specific reverse primers). Homoeolocus-specificity was confirmed by RT-PCR with the complete set of nullitetra lines. Each homoeolocus-specific PCR amplified the homoeologous product (i.e., gel bands were detected) in all samples except for the nullitetra lines lacking the corresponding homoeologous chromosome. Homoeologous sequences were confirmed by purification of PCR products using the QIAquick PCR purification kit (QIAGEN) and Sanger sequencing. We could amplify all three homoeoloci for all 10 genes. For 6 of these, all three homoeolocus-specific primer sets amplified a single homoeolocus with 100% specificity. These genes were selected for experimental verification of homoeolocus expression patterns. For the remaining 4 genes, it was not possible to design three completely specific primer sets, so their expression patterns were not verified. Details of primers and their sequences are listed in Additional file 12: Table S4C.

### Experimental validation of differential homoeologous expression patterns

Two independent approaches were used to experimentally validate differential homoeolocus expression patterns for 6 genes with all three homoeoloci amplified specifically (see Additional file 12: Table S4B). First, homoeolocus-specific PCR amplification products from the exponential amplification phase were run on a 1% agarose gel stained with ethidium bromide (Sigma) and band intensities were compared to quantify the expression levels of each homoeolocus (A, B, D). Second, all three homoeoloci amplified using common primers were purified by ethanol precipitation, ligated into pGEM-T Easy Vector and transformed into One Shot Top 10 competent cells (Invitrogen). Transformed cells were spread onto Luria-Broth agar plates containing ampicillin and incubated at 37°C overnight. 96 colonies were picked and cultured in 2 ml tubes at 37°C overnight. Plasmids were extracted using the Wizard SV 96 Plasmid kit (Promega) and cloned products were Sanger sequenced using T7 and SP6 primers. HSVs were used to assign the homoeolocus identity (A, B or D) of each clone, reflecting the relative proportions of the three homoeoloci *in vivo*.

### Availability of supporting data

The Illumina RNA-Seq data supporting the results of this article is available [62] in the NCBI sequence read archive [SRA: SRP028357].

## Abbreviations

HSV: Homoeologue-specific variant; Nullitetra: Nullisomic-tetrasomic.

## Competing interests

The authors declare that they have no competing interests.

## Authors’ contributions

NPH conceived of and designed the study, and helped to write the manuscript. LJL and EJB participated in the design and coordination of the research. LJL performed the RNA-Seq data analysis, experimental verification of genes with differential expression of homoeoloci by RT-PCR and PCR product cloning and wrote the paper. CB grew the wheat plants and collected samples for RNA-Sequencing. CJ performed some cloning experiments. AM contributed to the data analysis. All authors read and approved the final manuscript.

## Supplementary Material

Additional file 1: Table S1 Illumina RNA-sequencing data and read mapping statistics. This table summarizes the RNA-Seq datasets obtained from non-normalized cDNA libraries of wheat samples and the mapping results for alignment of reads to a partial reference transcriptome sequence using novoalign software. Click here for file

Additional file 2: Table S2 Origin of EST sequences. This table shows the species of origin for EST sequences used to construct the partial wheat reference transcriptome. Click here for file

Additional file 3: Figure S1 Length distribution for ESTs on wheat chromosome groups 1 and 5. This figure shows the length distribution for ESTs used to construct the partial wheat reference transcriptome. Click here for file

Additional file 4: Figure S2 Schematic illustration of homoeologue-specific variant (HSV) discovery in hexaploid bread wheat. This figure shows the bioinformatics pipeline used to identify and quantify homoeolocus-specific bread wheat sequences. Click here for file

Additional file 5: Figure S3 Qualitative gene expression in bread wheat. This figure shows the overlap between expressed genes in two biological replicates for shoot and root tissues. Click here for file

Additional file 6: Figure S4 Number of haplotypes observed within RNA-Seq reads mapped to regions of the reference sequence covered by homoeolocus-specific haplotypes. This figure shows the distribution of the number of haplotypes in 26,498 regions from 872 genes expressed from all three homoeologous chromosomes of group 1 (1A, 1B, 1D) or group 5 (5A, 5B, 5D) in roots. Click here for file

Additional file 7: Figure S5 Properties of transcript sequences for wheat genes on chromosome groups 1 and 5 showing either equal expression from all three homoeoloci (‘A=B=D’), differential expression of homoeoloci (‘DE’) or an unknown pattern of expression bias (‘U’) in shoots (white) or roots (blue). This figure shows how the total expression level (RPKM) and HSV (homoeologue-specific variant) density varies between genes with different patterns of expression from three homoeoloci (A, B and D). Click here for file

Additional file 8: Figure S6 Biased patterns of expression for homoeoloci on wheat group 1 and 5 chromosomes. This figure shows the contribution of transcripts from A, B and D homoeoloci for genes showing each possible pattern of differential expression. Click here for file

Additional file 9: Table S3 Significance of differential expression patterns for genes expressed from all three group 1 or 5 homoeoloci in shoots or roots. This table shows the significance level of differentially expressed genes in two biological replicates of shoots and roots. Click here for file

Additional file 10: Figure S7 Relationship between total gene expression level (RPKM) and variation in expression among three expressed homoeoloci. This figure shows the relationship between the total expression level of genes expressed from all three homoeoloci and the strength of differential expression bias. Click here for file

Additional file 11: Figure S8 Gel images depicting amplification of A, B and D homoeoloci of 6 genes by homoeologue-specific RT-PCR. This figure shows experimental confirmation of the homoeologue-specificity of A, B and D homoeoloci and the corresponding expression patterns of 6 group 1 or group 5 genes expressed from all three homoeoloci (A, B and D). Click here for file

Additional file 12: Table S4 Experimental validation of homoeolocus expression patterns shown by RNA-Seq of euploid wheat roots. This table details the experimental verification of homoeologue-specific variants (HSVs) and expression patterns for 6 genes expressed from all three homoeoloci (A, B and D). Click here for file

Additional file 13: Figure S9 Total expression level of wheat genes on group 1 and 5 chromosomes expressed from all three homoeoloci in both shoots and roots. This figure shows the total expression level of genes expressed from all three homoeoloci in both tissues is significantly higher in roots compared with shoots. Click here for file

Additional file 14: Table S5 Conflicting homoeolocus expression patterns between shoot and root organs of bread wheat. This table shows genes with different patterns of homoeolocus expression in shoot versus root tissues. Click here for file

Additional file 15: Table S6 Presence and expression of up to three homoeoloci for wheat group 1 genes. This table shows the number of genes physically present on and expressed from (in shoots or roots) each combination of group 1 homoeologous chromosomes. Click here for file

Additional file 16: Table S7 Dataset of assembled sequences for wheat group 1 chromosome arms. This table shows the dataset of assembled sequences for genes on wheat chromosomes 1A, 1B and 1D from Wicker et al. [52]. Click here for file

Additional file 17: Table S8 Comparison of homoeolocus expression between shoots and roots for genes on group 1 and 5 chromosomes. This table shows that most genes are expressed from the same homoeologous chromosomes in the two wheat tissues (shoots and roots). Click here for file
