# Supplementary material for: Patterns of homoeologous gene expression shown by RNA sequencing in hexaploid bread wheat
Source: BMC Genomics. 2014 Apr 11;15:276. doi: 10.1186/1471-2164-15-276 (PMC4023595; doi:10.1186/1471-2164-15-276)
Supplement: Additional file 2: Table S2 — Origin of EST sequences. This table shows the species of origin for EST sequences used to construct the partial wheat reference transcriptome. [file 1471-2164-15-276-S2.doc]

| Species | Number of ESTs | | |
| --- | --- | --- | --- |
|  | Chromosome 1 | Chromosome 5 | Total |
| *Triticum aestivum* | 932 | 1022 | 1,954 |
| *Triticum monococcum* | 86 | 79 | 165 |
| *Triticum turgidum* | 17 | 24 | 41 |
| *Secale cereale* | 52 | 75 | 127 |
| *Hordeum vulgare* | 1 | 0 | 1 |
| *Aegilops speltoides* | 35 | 45 | 80 |
| *Avena sativa* | 0 | 1 | 1 |
| Total | 1,123 | 1,246 | 2,369 |

**Supplemental Table S2. Origin of EST sequences.**

ESTs collected from a wide range of tissues, developmental stages and environmental conditions were acquired from the website of the U.S. Wheat EST project (<http://wheat.pw.usda.gov/cgi-bin/westsql/map_locus.cgi>).

The number of ESTs with each species of origin is shown for chromosomes 1 and 5.

1,954/2,369 ESTs (83%) are from hexaploid bread wheat (*T. aestivum*).

2,160/2,369 ESTs (91%) are from *Triticum* species.
