# Supplementary material for: Patterns of homoeologous gene expression shown by RNA sequencing in hexaploid bread wheat
Source: BMC Genomics. 2014 Apr 11;15:276. doi: 10.1186/1471-2164-15-276 (PMC4023595; doi:10.1186/1471-2164-15-276)
Supplement: Additional file 3: Figure S1 — Length distribution for ESTs on wheat chromosome groups 1 and 5. This figure shows the length distribution for ESTs used to construct the partial wheat reference transcriptome. [file 1471-2164-15-276-S3.doc]

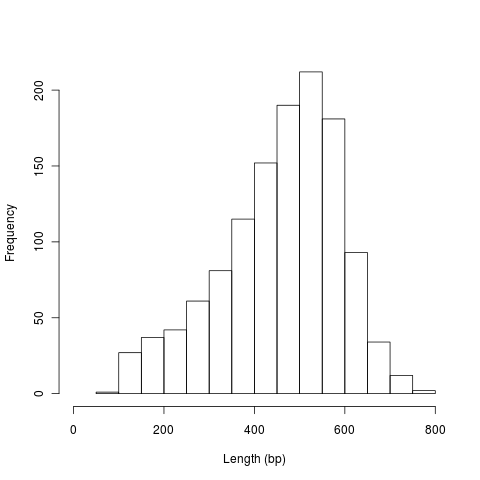


**Supplemental Figure S1. Length distribution for ESTs on wheat chromosomes 1 and 5.**

ESTs have mean length 455±133, n = 2,354.
