# Supplementary material for: Patterns of homoeologous gene expression shown by RNA sequencing in hexaploid bread wheat
Source: BMC Genomics. 2014 Apr 11;15:276. doi: 10.1186/1471-2164-15-276 (PMC4023595; doi:10.1186/1471-2164-15-276)
Supplement: Additional file 4: Figure S2 — Schematic illustration of homoeologue-specific variant (HSV) discovery in hexaploid bread wheat. This figure shows the bioinformatics pipeline used to identify and quantify homoeolocus-specific bread wheat sequences. [file 1471-2164-15-276-S4.doc]

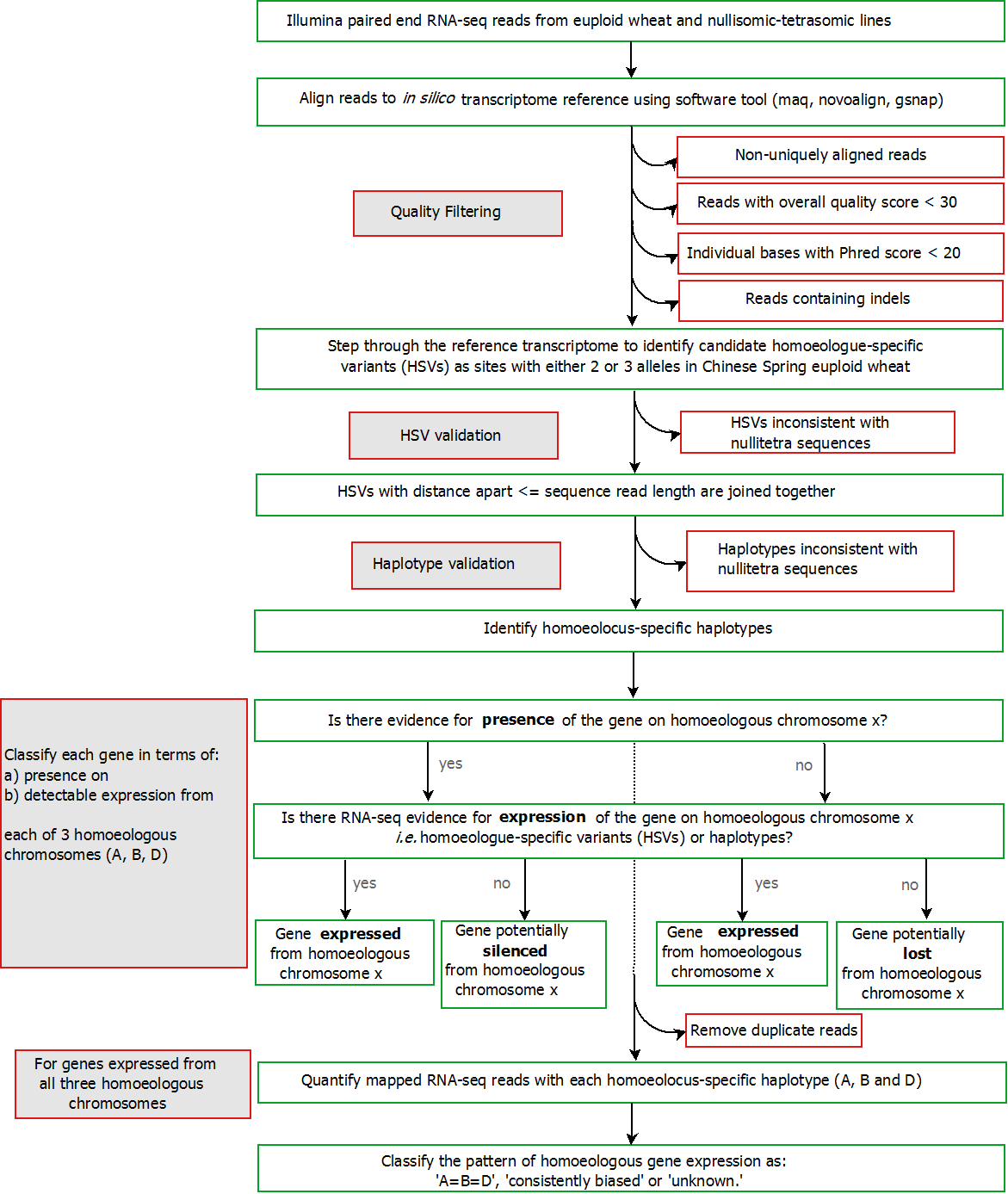


**Supplemental Figure S2. Schematic illustration of homoeologue-specific variant (HSV) discovery in hexaploid bread wheat.**

A custom bioinformatics workflow consisting of read mapping followed by quality filtering and homoeologue-specific variant (HSV) detection was implemented computationally using Perl and visually using the Integrative Genomics Viewer [1]. RNA-Seq reads were mapped to the reference sequence using each of three mapping softwares (maq, novoalign and gsnap) and passed through a number of quality filters. First, reads not aligning uniquely to a single location in the reference sequence were removed. Second, reads with a low mapping quality score (<30) were removed. Third, within each read, individual bases with a low quality score (Phred < 20, *i.e.* base call accuracy of 99% [2,3]) were ignored. Finally, any mapped reads containing nucleotide(s) insertions or deletions compared with the reference sequence were discarded. Locations within the reference sequence with 2 or 3 alleles (bases) in the filtered euploid wheat reads were identified as candidate HSVs, which were validated using sequences from the nullitetra lines of the corresponding chromosome group. Two-allele and three-allele HSVs were confirmed where the allele diagnostic (i.e., specific) for a particular homoeologue was present in all nullitetra lines except for those lacking the corresponding homoeologous chromosome. HSVs co-located within a region of sequence less than the length of individual sequence reads (51 base pairs) were combined to produce haplotypes and the homoeolocus-specificity of haplotypes was confirmed. Deletion bin-mapping data [4,5] and chromosome arm-specific assemblies for group 1 homoeologues [6] were used together with our RNA-Seq datasets to infer the presence and expression of each gene across the three possible homoeologous chromosomes (A, B and D). For genes unequivocally expressed from all three homoeologous chromosomes, duplicate reads were removed and the homoeolocus specific haplotypes were quantified. The expression pattern could be classified as either equal expression from all three homoeoloci ('A=B=D'), differential expression of homoeoloci ('consistently biased') or as 'unknown' where no consistent and significant pattern was detected.

1. Robinson JT, Thorvaldsdóttir H, Winckler W, Guttman M, Lander ES, Getz G, Mesirov JP: **Integrative genomics viewer.** *Nat Biotech* 2011, **29**:24–6.
2. Li H, Handsaker B, Wysoker A, Fennell T, Ruan J, Homer N, Marth G, Abecasis G, Durbin R: **The Sequence Alignment/Map format and SAMtools.** *Bioinformatics* 2009, **25**: 2078-2079.
3. Cock PJA, Fields CJ, Goto N, Heuer ML, Rice PM: **The Sanger FASTQ file format for sequences with quality scores, and the Solexa/Illumina FASTQ variants.** *Nucleic Acids Res.* 2010, **38**: 1767–1771.
4. Qi L, Echalier B, Friebe B, Gill BS: **Molecular characterization of a set of wheat deletion stocks for use in chromosome bin mapping of ESTs.** *Funct Integr Genomics* 2003, **3**:39–55.
5. Qi LL, Echalier B, Chao S, Lazo GR, Butler GE, Anderson OD, Akhunov ED, Dvorák J, Linkiewicz AM, Ratnasiri A, Dubcovsky J, Bermudez-Kandianis CE, Greene RA, Kantety R, La Rota CM, Munkvold JD, Sorrells SF, Sorrells ME, Dilbirligi M, Sidhu D, Erayman M, Randhawa HS, Sandhu D, Bondareva SN, Gill KS, Mahmoud AA, Ma X-F, Miftahudin, Gustafson JP, Conley EJ, et al.: **A chromosome bin map of 16,000 expressed sequence tag loci and distribution of genes among the three genomes of polyploid wheat.** *Genetics* 2004, **168**:701–12.
6. Wicker T, Mayer KFX, Gundlach H, Martis M, Steuernagel B, Scholz U, Simková H, Kubaláková M, Choulet F, Taudien S, Platzer M, Feuillet C, Fahima T, Budak H, Dolezel J, Keller B, Stein N: **Frequent gene movement and pseudogene evolution is common to the large and complex genomes of wheat, barley, and their relatives.** *Plant Cell* 2011, **23**:1706–18.
