# Supplementary material for: Patterns of homoeologous gene expression shown by RNA sequencing in hexaploid bread wheat
Source: BMC Genomics. 2014 Apr 11;15:276. doi: 10.1186/1471-2164-15-276 (PMC4023595; doi:10.1186/1471-2164-15-276)
Supplement: Additional file 5: Figure S3 — Qualitative gene expression in bread wheat. This figure shows the overlap between expressed genes in two biological replicates for shoot and root tissues. [file 1471-2164-15-276-S5.doc]

**
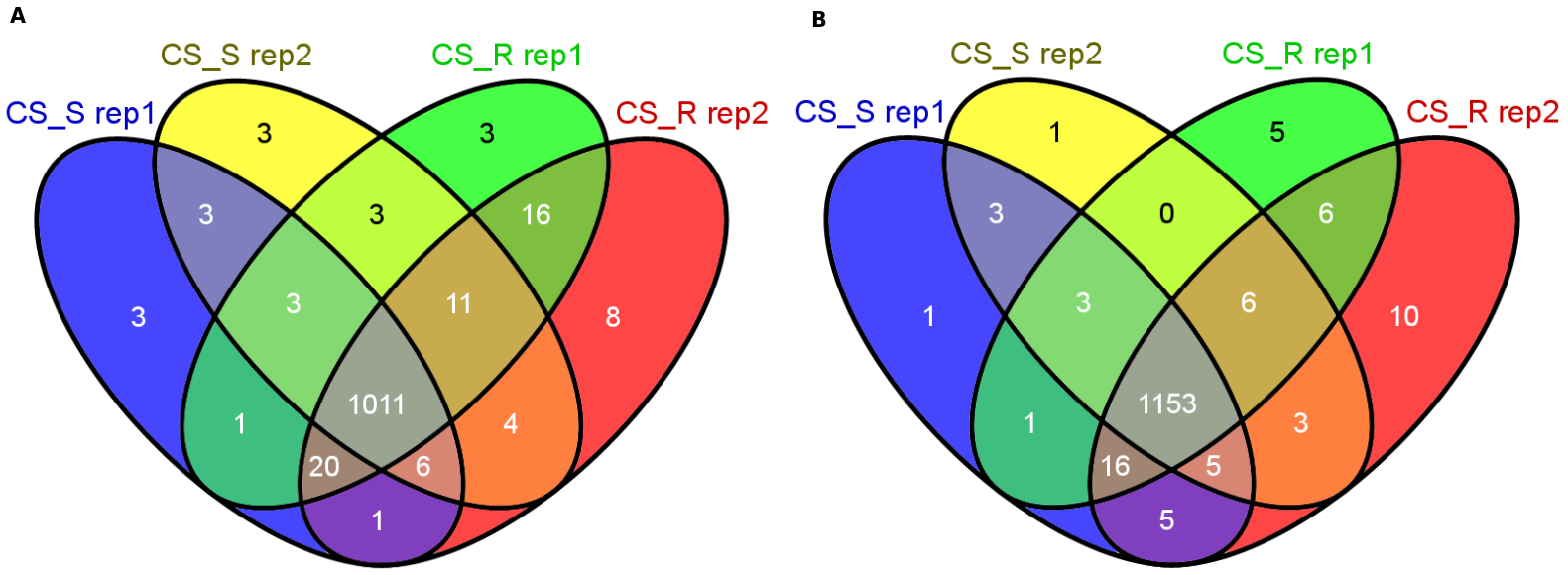
**

**Supplemental Figure S3. Qualitative gene expression in bread wheat.**

Venn diagrams showing overlap of genes on chromosome group 1 **(A)** or group 5 **(B)** called as expressed in two biological replicates of Chinese Spring shoot (CS_S) and root (CS_R) tissues.
