# Supplementary material for: Patterns of homoeologous gene expression shown by RNA sequencing in hexaploid bread wheat
Source: BMC Genomics. 2014 Apr 11;15:276. doi: 10.1186/1471-2164-15-276 (PMC4023595; doi:10.1186/1471-2164-15-276)
Supplement: Additional file 6: Figure S4 — Number of haplotypes observed within RNA-Seq reads mapped to regions of the reference sequence covered by homoeolocus-specific haplotypes. This figure shows the distribution of the number of haplotypes in 26,498 regions from 872 genes expressed from all three homoeologous chromosomes of group 1 (1A, 1B, 1D) or group 5 (5A, 5B, 5D) in roots. [file 1471-2164-15-276-S6.doc]

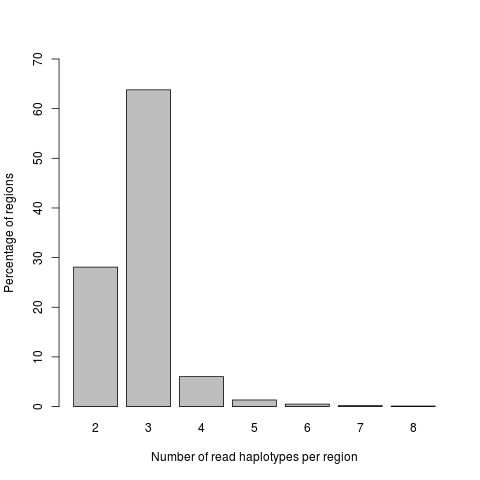


**Supplemental Figure S4. Number of haplotypes observed within RNA-Seq reads mapped to regions of the reference sequence covered by homoeolocus-specific haplotypes.**

n = 26,498 regions from n = 872 genes expressed from all three homoeologous chromosomes of group 1 (1A, 1B, 1D) or group 5 (5A, 5B, 5D) in roots.
