# Supplementary material for: Patterns of homoeologous gene expression shown by RNA sequencing in hexaploid bread wheat
Source: BMC Genomics. 2014 Apr 11;15:276. doi: 10.1186/1471-2164-15-276 (PMC4023595; doi:10.1186/1471-2164-15-276)
Supplement: Additional file 7: Figure S5 — Properties of transcript sequences for wheat genes on chromosome groups 1 and 5 showing either equal expression from all three homoeoloci (‘A=B=D’), differential expression of homoeoloci (‘DE’) or an unknown pattern of expression bias (‘U’) in shoots (white) or roots (blue). This figure shows how the total expression level (RPKM) and HSV (homoeologue-specific variant) density varies between genes with different patterns of expression from three homoeoloci (A, B and D). [file 1471-2164-15-276-S7.doc]

**A**

**
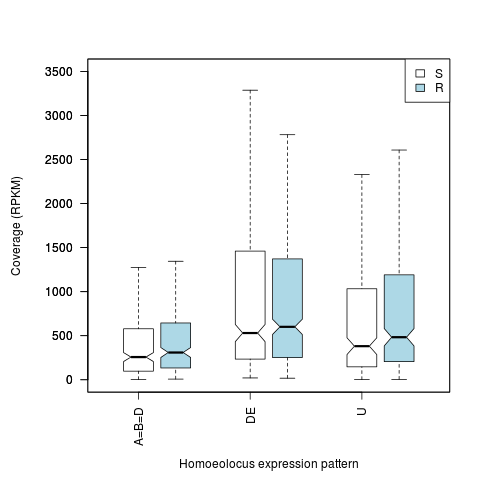
**

**B**

**
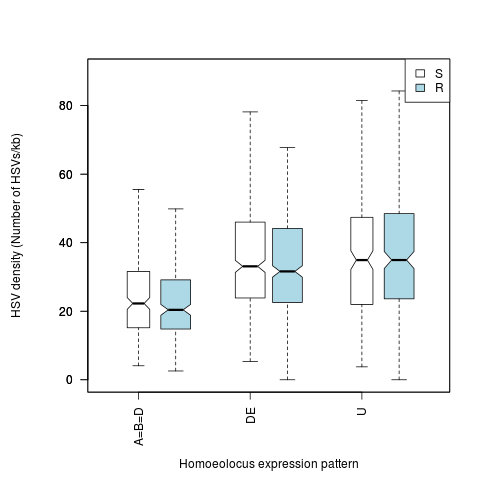
**

**C**

**
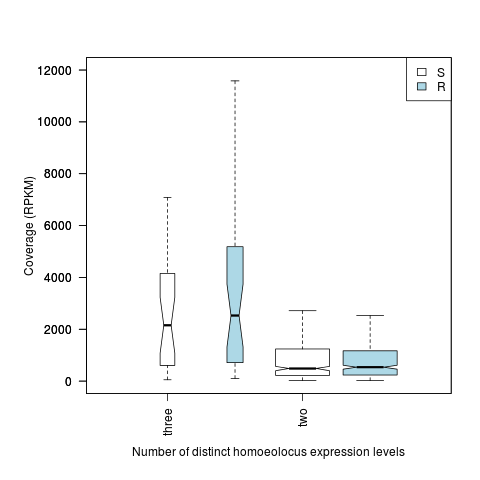
**

**Supplemental Figure S5. Properties of transcript sequences for wheat genes on chromosome groups 1 and 5 showing either equal expression from all three homoeoloci (‘A=B=D’), differential expression of homoeoloci (‘DE’) or an unknown pattern of expression bias (‘U’) in shoots (white) or roots (blue).**

For genes in group U (unknown), a consistent and significant pattern of differential expression of homoeoloci could not be detected. Genes with significant differential expression (DE) show a consistent pattern (between biological replicates and along the sequence length) and pass a Bonferroni-corrected threshold of *p* < .05. Homoeoloci are either expressed at three distinct expression levels (e.g., ‘A>B>D’) or two distinct levels, i.e., two homoeoloci expressed at the same level (e.g., ‘A=B>D’).

**A)** Total expression level from all three homoeoloci given as RPKM (reads per kb per million aligned reads).

RPKM of genes expressed in shoots is distributed as 760.40±2778.07 ('A=B=D' group, n=225), 1283.08±2979.91 ('DE' group, n=391) and 1005.69±1777.22 ('U' group, n=217).

RPKM of genes expressed in roots is distributed as 973.71±4905.10 ('A=B=D' group, n=206), 1561.83±3510.28 ('DE' group, n=417) and 1256.94±3409.32 ('U' group, n=249).

Genes with ‘A=B=D’ have a significantly lower expression level compared with ‘DE’ genes (Mann Whitney U test, *p* < .001).

**B)** HSV (homoeologue-specific variant) density.

For genes expressed in shoots is distributed as 24.85±12.76 ('A=B=D' group, n=225), 35.15±15.62 ('DE' group, n=391) and 37.03±19.34 ('U' group, n=217).

For genes expressed in roots is distributed as 23.06±11.77 ('A=B=D' group, n=206), 33.93±15.47 ('DE' group, n=417) and 36.74±18.61 ('U' group, n=249).

Genes with ‘A=B=D’ have a significantly lower HSV density compared with ‘DE’ genes (Mann Whitney U test, *p* < .001).

**C)** Total expression level from all three homoeoloci given as RPKM (reads per kb per million aligned reads) for genes with homoeoloci expressed at three distinct levels (group 'three') or at two distinct levels (group 'two'). The width of each box is proportional to the number of genes.

The RPKM of genes expressed in shoots is distributed as 3764.78±5977.01 ('three' group, n=27) and 1099.50±1621.63 ('two' group, n=364).

The RPKM of genes expressed in roots is distributed as 4822.00±8384.96 ('three' group, n=34) and 1272.41±2513.00 ('two' group, n=383).

Genes with ‘A=B=D’ have a significantly lower HSV density compared with ‘DE’ genes (Mann Whitney U test, *p* < .001).
