# Supplementary material for: Patterns of homoeologous gene expression shown by RNA sequencing in hexaploid bread wheat
Source: BMC Genomics. 2014 Apr 11;15:276. doi: 10.1186/1471-2164-15-276 (PMC4023595; doi:10.1186/1471-2164-15-276)
Supplement: Additional file 8: Figure S6 — Biased patterns of expression for homoeoloci on wheat group 1 and 5 chromosomes. This figure shows the contribution of transcripts from A, B and D homoeoloci for genes showing each possible pattern of differential expression. [file 1471-2164-15-276-S8.doc]

**
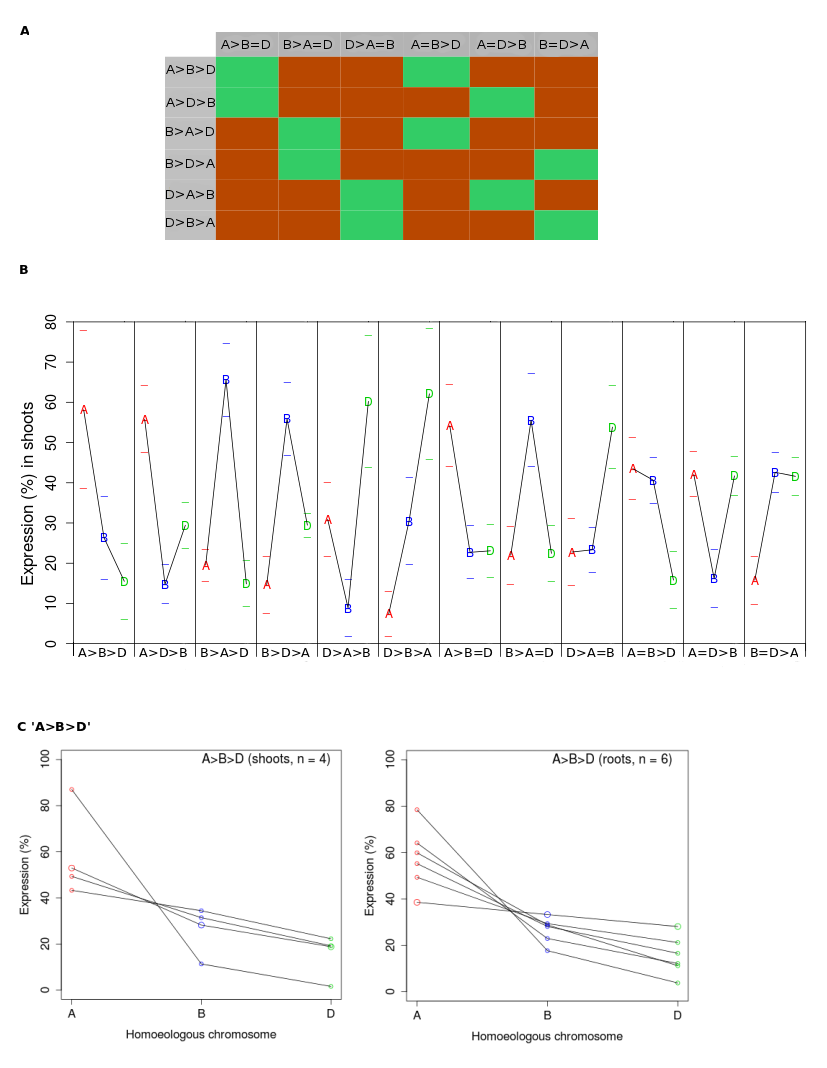
**

**
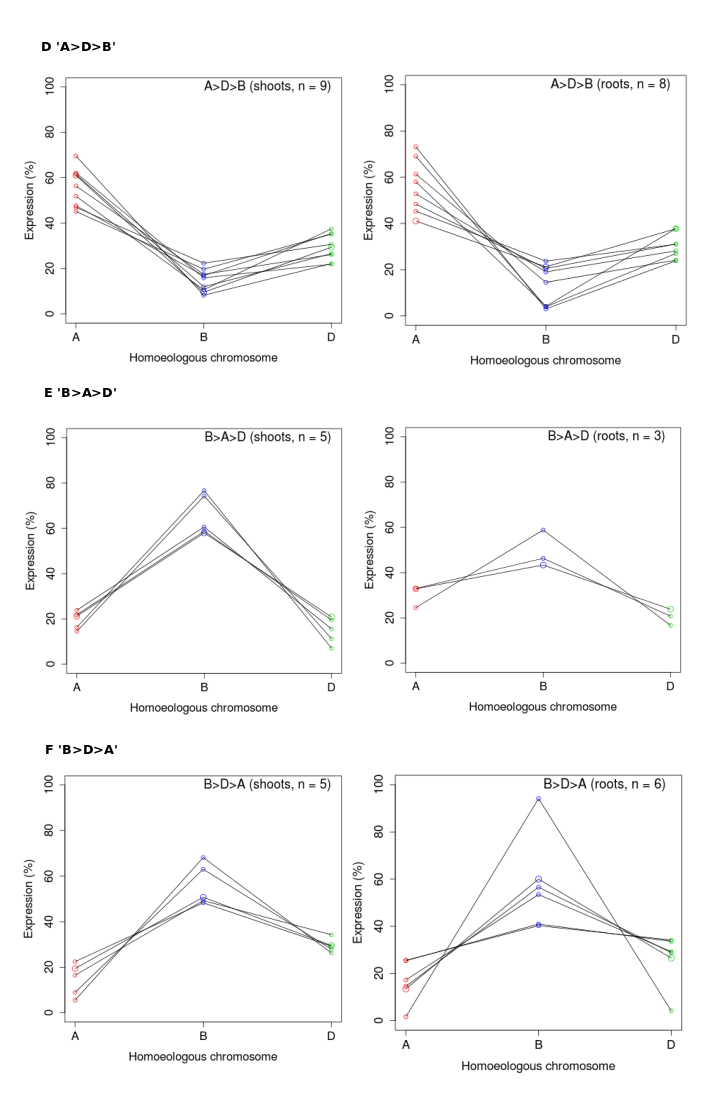
**

**
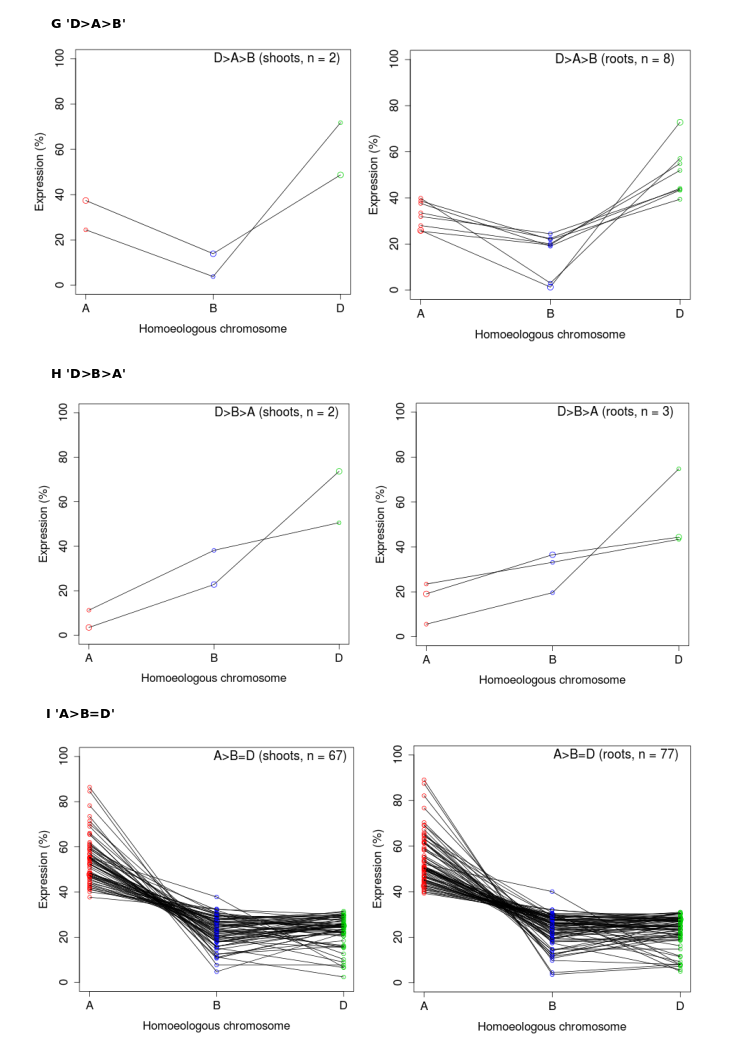
**

**
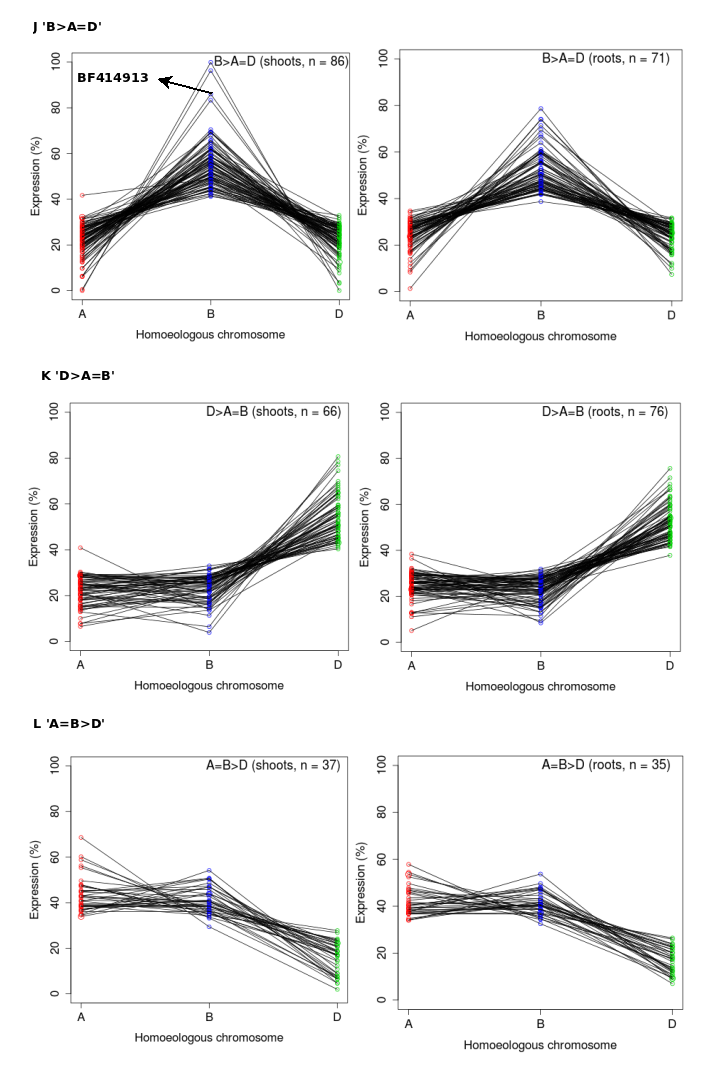
**

**
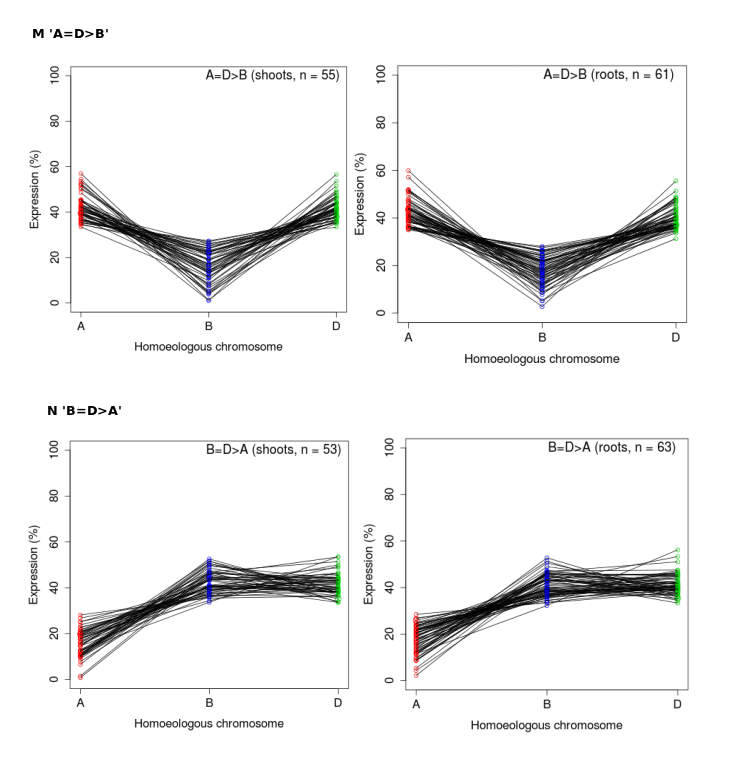
**

**Supplemental Figure S6. Biased patterns of expression for homoeoloci on wheat group 1 and 5 chromosomes.**

**(A)** Compatibility among twelve distinct patterns of differential homoeolocus expression. Each cell shows whether the corresponding conclusions are consistent (green) or inconsistent (red). There are six patterns (rows) distinguishing three distinct levels of expression, each of which ranks the expression levels of the three homoeoloci and is consistent with two of the six patterns (columns) distinguishing two levels of expression.

**(B)** The mean percentage of total transcripts arising from A (red), B (blue) and D (green) homoeoloci for 12 distinct patterns of differential expression in shoots. Error bars are given at one standard deviation above and below the mean.

**(C)-(N)** The mean percentage of transcripts arising from A, B and D homoeoloci for individual genes showing each of the 12 patterns of differential expression in shoots or roots.
