# Supplementary material for: Patterns of homoeologous gene expression shown by RNA sequencing in hexaploid bread wheat
Source: BMC Genomics. 2014 Apr 11;15:276. doi: 10.1186/1471-2164-15-276 (PMC4023595; doi:10.1186/1471-2164-15-276)
Supplement: Additional file 10: Figure S7 — Relationship between total gene expression level (RPKM) and variation in expression among three expressed homoeoloci. This figure shows the relationship between the total expression level of genes expressed from all three homoeoloci and the strength of differential expression bias. [file 1471-2164-15-276-S10.doc]

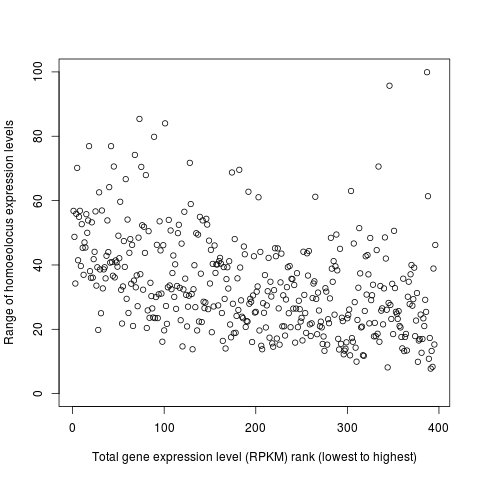

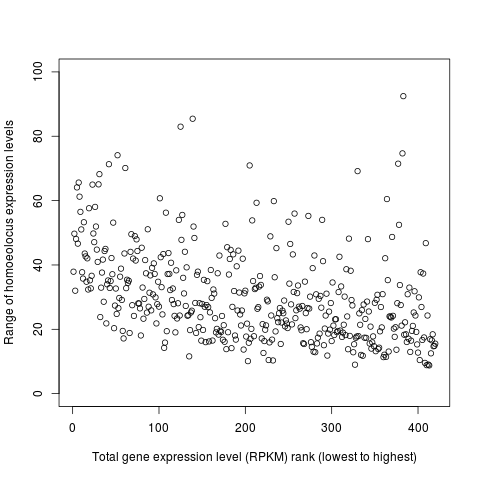
**A B**

**Supplemental Figure S7. Relationship between total gene expression level (RPKM) and variation in expression among three expressed homoeoloci.**

Genes expressed from all three homoeoloci are ranked from lowest to highest according to their total expression level (RPKM) in shoots **(A)** or roots **(B)**. Variation in expression among the three homoeoloci is given as the range of the average percentage of reads arising from the three homoeoloci *i.e.* (max. homoeolocus % – min. homoeolocus %).
