# Supplementary material for: Patterns of homoeologous gene expression shown by RNA sequencing in hexaploid bread wheat
Source: BMC Genomics. 2014 Apr 11;15:276. doi: 10.1186/1471-2164-15-276 (PMC4023595; doi:10.1186/1471-2164-15-276)
Supplement: Additional file 11: Figure S8 — Gel images depicting amplification of A, B and D homoeoloci of 6 genes by homoeologue-specific RT-PCR. This figure shows experimental confirmation of the homoeologue-specificity of A, B and D homoeoloci and the corresponding expression patterns of 6 group 1 or group 5 genes expressed from all three homoeoloci (A, B and D). [file 1471-2164-15-276-S11.doc]

**A**

**
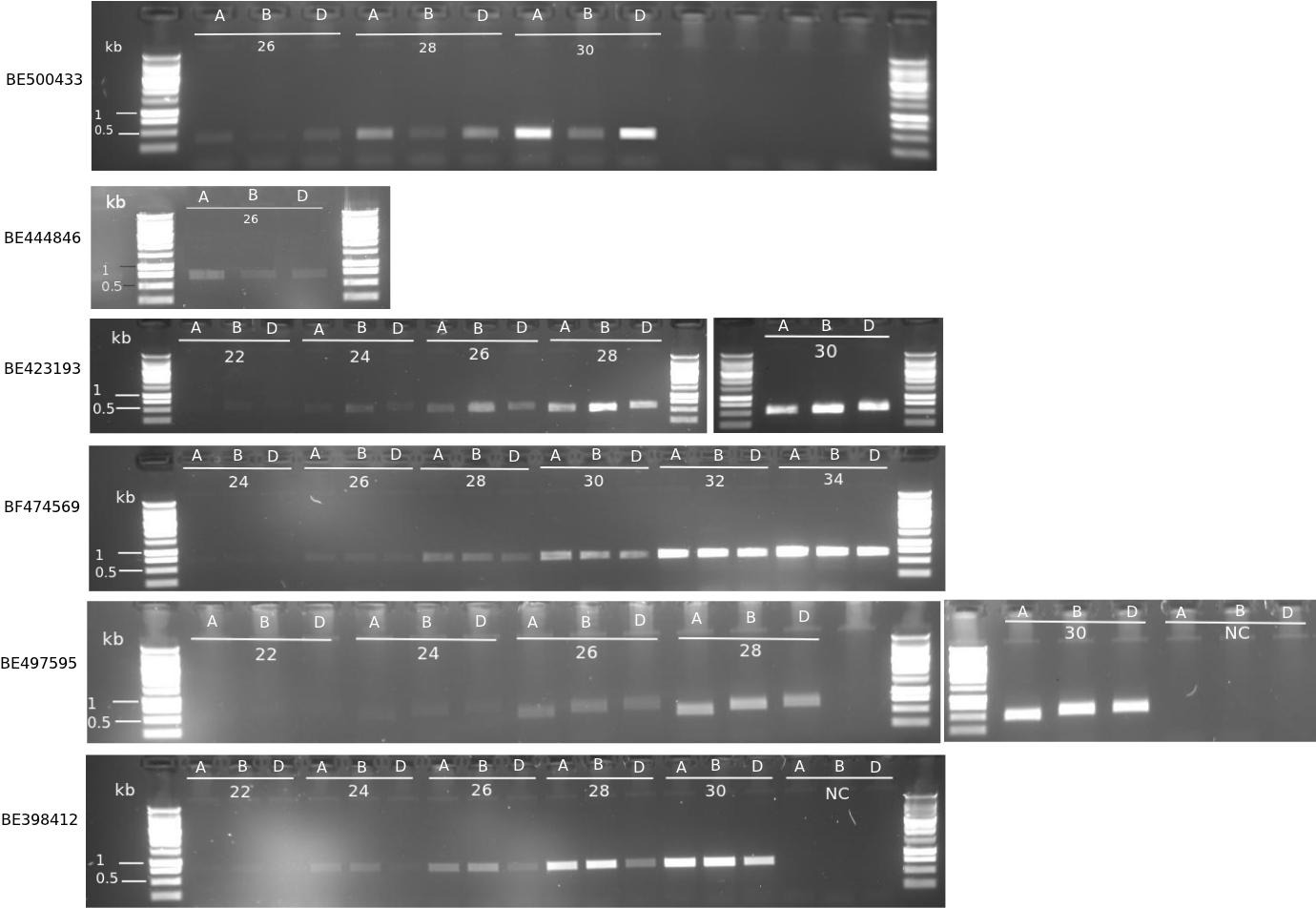
**

**B**

**
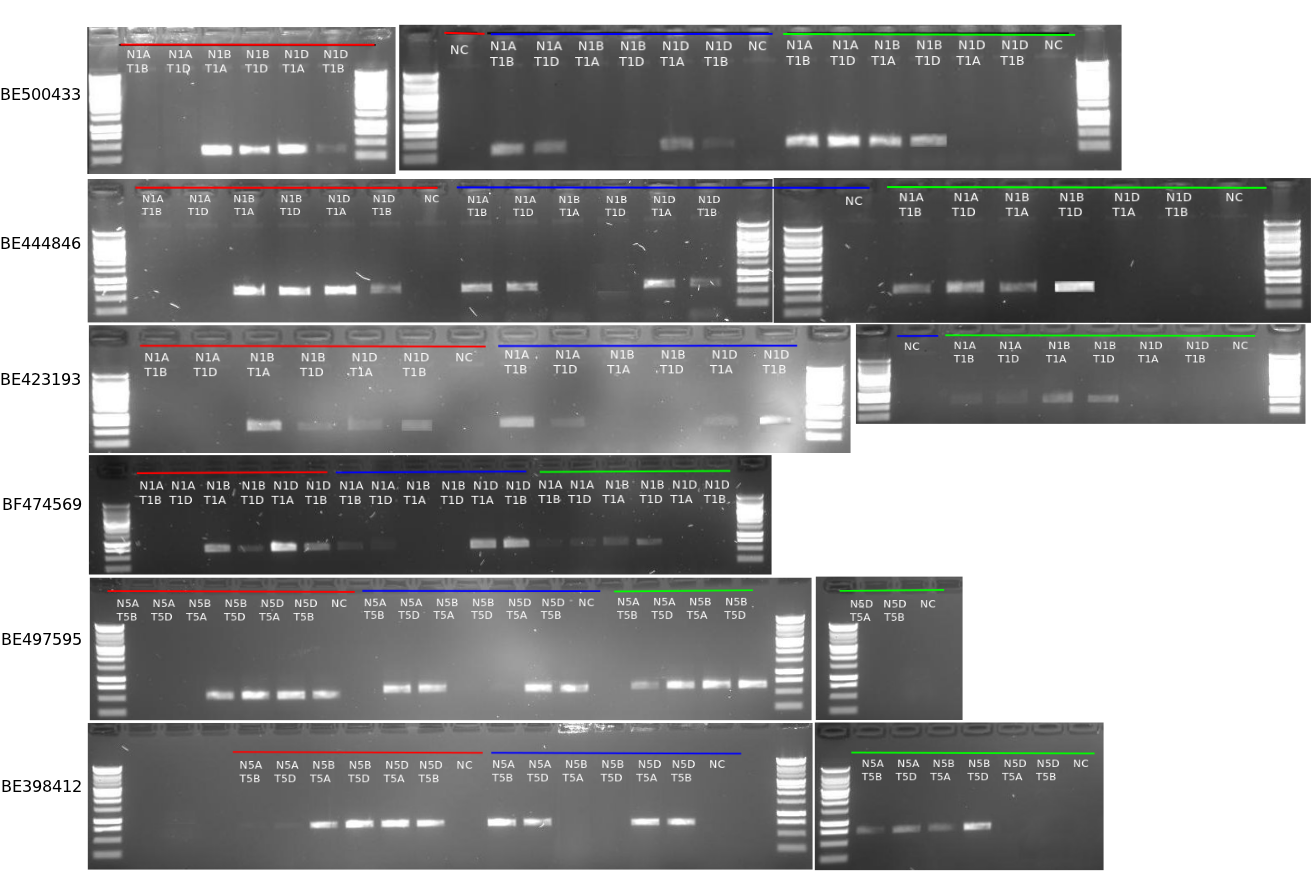
**

**Supplemental Figure S8. Gel images depicting amplification of A, B and D homoeoloci of 6 genes by homoeologue-specific RT-PCR.**

The 6 verified genes are: BE500433, BE444846, BE423193, BF474569, BE497595 and BE398412, as detailed in **Additional file 12:** **Table S4B**. All PCR amplifications were performed using cDNA created from the same root RNA samples used for RNA-seq. **(A)** Amplification of A, B and D homoeologues from Chinese Spring euploid wheat, with the number of PCR cycles indicated. **(B)** Amplification of A (red), B (blue) and D (green) homoeologues from all nullitetra lines using 30 PCR cycles. NC, negative control.
