# Supplementary material for: Patterns of homoeologous gene expression shown by RNA sequencing in hexaploid bread wheat
Source: BMC Genomics. 2014 Apr 11;15:276. doi: 10.1186/1471-2164-15-276 (PMC4023595; doi:10.1186/1471-2164-15-276)
Supplement: Additional file 12: Table S4 — Experimental validation of homoeolocus expression patterns shown by RNA-Seq of euploid wheat roots. This table details the experimental verification of homoeologue-specific variants (HSVs) and expression patterns for 6 genes expressed from all three homoeoloci (A, B and D). [file 1471-2164-15-276-S12.doc]

A

| Chromosome:  gene name | RNA-Seq  expression  pattern | TIGR contig corresponding to homoeolocus | | | Number of homoeoloci amplified with 100% specificity / 3 |
| --- | --- | --- | --- | --- | --- |
|  |  | A | B | D |  |
| **chr1:*BF474569*** | A>B=D | TC380684 | TC375032 | TC381867 | **3** |
| **chr1:*BE500433*** | A>D>B | TC373860 | TC381864 | TC397870 | **3** |
| **chr1:*BE444846*** | A>D>B | TC454582 | TC401875 | TC392843 | **3** |
| **chr1:*BE423193*** | B>D>A | TC407376 | TC411715 | TC412567 | **3** |
| **chr5:*BE497595*** | B>A=D | TC385339 | TC383307 | TC391961 | **3** |
| **chr5:*BE398412*** | B>A=D | TC370004 | TC368662 | TC368678 | **3** |
| chr5:*BE445396* | D>B>A | TC394123 | TC381820 | TC384956 | 2 |
| chr1:*BF200980* | A>D>B | TC385976 | TC438373 | TC438255 | 2 |
| chr1:*BE490592* | B>A=D | TC419976 | TC374505 | TC397255 | 1 |
| chr5:*BE637097* | A>B=D | TC388737 | TC438609 | TC392569 | 0 |

**B**

| Chromosome:  gene name | Function | # HSVs detected (missed) | RNA-Seq  pattern | Strength (%) of expression from each homoeolocus from RNA-Seq reads (mean±stdev) | | | Validation I: RT-PCR  Mean band intensity (amplicon length) for homoeolocus | | | Validation II: bacterial cloning  Number of clones (%) with homoeolocus identity | | | Validation II:  P-value | Patterns Validated (I/II) |
| --- | --- | --- | --- | --- | --- | --- | --- | --- | --- | --- | --- | --- | --- | --- |
|  |  |  |  | A | B | D | A | B | D | A | B | D |  |  |
| 1:*BE444846* | Hypothetical | 40 (2) | A>D>B | 41.05±0.27 | 21.24±1.22 | 37.71±0.95 | 18.1 (631) | 5.6 (644) | 7.6 (642) | 62 (71.3) | 18 (20.7) | 7  (8.0) | 2.07x10^-13^ | - /A>B, A>D |
| 1:*BE423193* | Ribosomal protein L36 | 10 (7) | B>D>A | 25.58±0.12 | 40.27±0.21 | 34.16±0.26 | 16.0 (541) | 41.4 (547) | 19.2 (593) | 21 (22.8) | 50 (54.4) | 21 (22.8) | 1.07x10^-4^ | B,D>A / B>A,D |
| 1:*BF474569* | Universal stress protein | 19 (1) | A>B=D | 41.52±0.54 | 28.75±1.13 | 29.73±0.92 | 19.4 (683) | 15.5 (685) | 11.8 (672) | n/a | n/a | n/a | n/a | A>D / n/a |
| 1:*BE500433* | Legumin-like | 8 (2) | A>D>B | 52.82±2.76 | 19.06±2.61 | 28.12±1.33 | 31.1 (350) | 6.2 (350) | 28.7 (350) | 6**^a^**  (7.2) | 22 (26.5) | 55 (66.3) | 1.69x10^-4^,**^b^** | A>D>B / D>B |
| 5:*BE497595* | thylakoidal peptidase | 26 (0) | B>A=D | 34.07±10.08 | 44.61±9.72 | 31.72±13.10 | 10.1 (512) | 13.7 (599) | 7.7 (635) | 31 (35.2) | 28 (31.8) | 29 (33.0) | > 0.05 | B>A / - |
| 5:*BE398412* | EREB domain | 15 (14) | B>A=D | 23.21±2.59 | 52.16±3.72 | 24.63±3.40 | 26.2 (726) | 36.7 (727) | 10.8 (728) | 28 (30.4) | 45 (48.9) | 19 (20.7) | 3.39x10^-3^ | B>A / B>A>D^c^ |

C

| Gene name  (EST length) | Homoeoloci amplified | TIGR contig | Forward primer | Reverse primer | Amplicon length (bp) | PCR annealing temp (ºc) |
| --- | --- | --- | --- | --- | --- | --- |
| BE444846 | A | TC454582 | CGACCTCCTCAAGTTCCTC | CATTCGAATCCCCTCAACAG | 631 | 59 |
| (528) | B | TC401875 |  | GCACAGCAACAGCATTCAGAG | 644 |  |
|  | D | TC392843 |  | GCACAGCAGCAGCATTCTC | 642 |  |
|  | A, B, D | - |  | TATTGTTACTGCGTCGGGATG | 489 |  |
| BE423193 | A | TC407376 | CCCAAGTCGGGCCTCTTC | AATGGCAGATGGAATCAAGAC | 541 | 60 |
| (506) | B | TC411715 |  | TATGAATGGCAGATGGAATCG | 547 |  |
|  | D | TC412567 |  | CACAGAAATTACGCACACATACG | 593 |  |
|  | A, B, D | - |  | GCAAACGATGCATTGCAAG | 489/489/490 |  |
| BF474569 | A | TC380684 | GACTTCTCGGAGGGGAGC | CGACTCATGGGGCAAAAC | 683 | 62 |
| (624) | B | TC375032 |  | GCAGCACGGCCTGACTCAG | 685 |  |
|  | D | TC381867 |  | GCACGGACTGACTCATGGA | 672 |  |
| BE500433 | A | TC373860 | ATGTCGCTCCCCAGCTACTC | CTGGAGACGAGCTTGGCG | 350 | 63 |
| (147) | B | TC381864 |  | CTGGAGACGAGCTTGGCA |  |  |
|  | D | TC397870 |  | CTGGAGACGAGCTTGGCC |  |  |
|  | A, B, D | - |  | GCAGTTAAGCGCCATGTCC | 441 |  |
| BE497595 | A | TC385339 | CTTCAGGCATTGGGCTACAG | GGCAGGGGAAAAACTGAGTC | 512 | 60 |
| (524) | B | TC383307 |  | TGCCATGGATGCTCATTCA | 599 |  |
|  | D | TC391961 |  | GCCAAACGCCCAAACATAAC | 635 |  |
|  | A, B, D | - |  | GAGCCAGACCTTTGGTTACAG | 572/574/575 |  |
| BE398412 | A | TC370004 | GCTCCAACTGTTCAGAAGTC | GAAACATAGCAGATGCAACCAG | 726 | 59 |
| (606) | B | TC368662 |  | CAAGCATAACAGATGCAACCT | 727 |  |
|  | D | TC368678 |  | CGAAACATAGCAGATGCAATCAA | 728 |  |
|  | A, B, D | - |  | ACATGCAAGGGACATTTGTG | 659/661/661 |  |

**Supplemental Table S4. Experimental validation of homoeolocus expression patterns shown by RNA-Seq of euploid wheat roots.**

**(A)** 10 genes expressed differentially from A, B and D homoeoloci of group 1 or group 5 chromosomes. For each gene we amplified all three homoeoloci by RT-PCR using homoeolocus-specific primers and confirmed their sequences using Sanger sequencing. For 6 of these genes (shown in bold), all three sets of homoeolocus-specific primers were 100% specific for amplifying the target homoeolocus from euploid wheat, while for the remaining four genes, one or more sets of primers were not 100% specific and amplified 2 or even all three homoeoloci. The set of 6 genes with specific primers sets were selected for experimental verification of their expression patterns. First, we confirmed the homoeolocus-specificity of each homoeolocus using the complete set of nullitetra lines. Each homoeolocus-specific PCR amplified the homoeologous product (*i.e.* gel bands were detected and their sequences confirmed by Sanger sequencing) in all samples except for the nullitetra lines lacking the corresponding homoeologous chromosome (see also **Additional file 11: Figure S8**). Next, we confirmed the expression patterns by methods I and II as follows.

**(B)** The number of HSVs detected is the number of homoeologue-specific variants (HSVs) in euploid wheat shown by nullitetra analysis, while the number of HSVs missed includes those shown by Sanger sequencing of homoeologous products, but missed in the nullitetra analysis. The strength of differential homoeolocus expression in the RNA-Seq data from euploid wheat roots is calculated as a weighted average of the percentage of reads arising from each homoeolocus in each haplotyped region of the sequence.

The expression patterns validated by methods I (semi-quantitative RT-PCR) or II (bacterial cloning) are indicated separately, while '-' indicates that no patterns were validated and 'n/a' indicates validation experiments not performed. For 5/6 genes the amplicon lengths differed for one or more homoeoloci because it was impossible to design three homoeolocus specific primers within a single sequence region. All expression differences between homoeoloci were confirmed for each gene, though in this table we indicate expression patterns as validated only in the absence of possible confounding by amplicon length. P-values are given for a chi square goodness of fit test against a 1:1:1 expression ratio.

Method II confirmed the differential expression patterns for 4/5 genes. For the unconfirmed gene (*BE497595*), we observed equal numbers of A, B and D clones. More clones are probably required to identify mild differential expression, such as results from the RNA-Seq data that <50% of total gene expression arose from the upregulated B homoeolocus.

^a^For *BE500433* we observed an unexpectedly low number of A homoeolocus clones, in marked contrast to the RNA-Seq results, which showed 'A>D>B'. There is no obvious explanation for this discrepancy. We were, however, able to confirm 'D>B' for the other two homoeoloci of this gene.

^b^P-value for comparison of the number of B and D clones with a 1:1 expectation.

^c^For *BE398412* the three homoeoloci are distinguished at three distinct levels ('B>A>D') by both methods of experimental verification, while the RNA-Seq data confirmed only 'B>A=D'. The RNA-Seq data also supported the 'A>D' pattern, though the moderate read depth and mild strength of differential homoeolocus expression limited the statistical power to uncover the actual differential expression pattern ('B>A>D').

**(C)** RT-PCR primers used to verify homoeolocus expression patterns. All primer sequences are given in 5' to 3' orientation. Amplicon length is given for A/B/D products for primers that amplify all three homoeoloci.
