# Supplementary material for: Patterns of homoeologous gene expression shown by RNA sequencing in hexaploid bread wheat
Source: BMC Genomics. 2014 Apr 11;15:276. doi: 10.1186/1471-2164-15-276 (PMC4023595; doi:10.1186/1471-2164-15-276)
Supplement: Additional file 13: Figure S9 — Total expression level of wheat genes on group 1 and 5 chromosomes expressed from all three homoeoloci in both shoots and roots. This figure shows the total expression level of genes expressed from all three homoeoloci in both tissues is significantly higher in roots compared with shoots. [file 1471-2164-15-276-S13.doc]

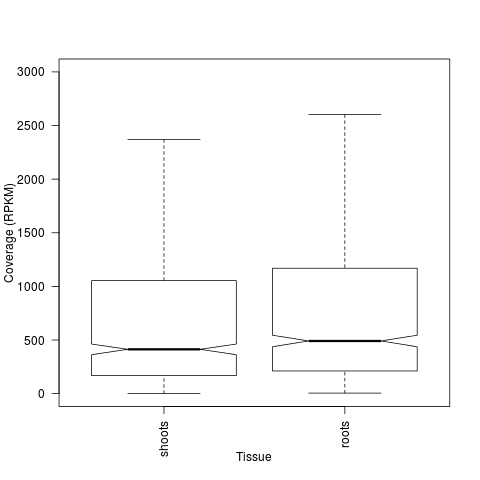


**Supplemental Figure S9. Total expression level of wheat genes on group 1 and 5 chromosomes expressed from all three homoeoloci in both shoots and roots.**

Total expression level from all three homoeoloci as RPKM (reads per kb per million mapped reads).

Expression level in shoots is distributed as 1082.08±2369.87 (n=787).

Expression level in roots is distributed as 1415.58±4046.65 (n=787).

Average expression level is significantly higher in roots compared with shoots (Mann-Whitney U test, *p* = .014).
