# Supplementary material for: Patterns of homoeologous gene expression shown by RNA sequencing in hexaploid bread wheat
Source: BMC Genomics. 2014 Apr 11;15:276. doi: 10.1186/1471-2164-15-276 (PMC4023595; doi:10.1186/1471-2164-15-276)
Supplement: Additional file 16: Table S7 — Dataset of assembled sequences for wheat group 1 chromosome arms. This table shows the dataset of assembled sequences for genes on wheat chromosomes 1A, 1B and 1D from Wicker et al. [52]. [file 1471-2164-15-276-S16.doc]

| Chromosome arm | Number of sequences | Totals |
| --- | --- | --- |
| Colinear sequences | | |
| 1AL | 1,359 |  |
| 1BL | 1,382 |  |
| 1DL | 1,427 |  |
| 1AS | 403 |  |
| 1BS | 461 | 5,032 |
| Non-colinear sequences | | |
| 1AL | 2,114 |  |
| 1BL | 2,309 |  |
| 1DL | 1,759 |  |
| 1AS | 946 |  |
| 1BS | 2,253 | 9,381 |
| Grand total | | 14,413 |

**Supplemental Table S7. Dataset of assembled sequences for wheat group 1 chromosome arms.**

Colinear sequences are genes that have their closest homologs in the Triticeae group 1 syntenic regions of *Brachpodium*, rice and/or sorghum genomes, while non-colinear sequences have their closest homologs outside those regions. No sequences were available for chromosome 1DS due to rearrangements in the corresponding ditelosomic line [1].

1. Wicker T, Mayer KFX, Gundlach H, Martis M, Steuernagel B, Scholz U, Simková H, Kubaláková M, Choulet F, Taudien S, Platzer M, Feuillet C, Fahima T, Budak H, Dolezel J, Keller B, Stein N: **Frequent gene movement and pseudogene evolution is common to the large and complex genomes of wheat, barley, and their relatives.** *Plant Cell* 2011, **23**:1706–1718.
